# Differential processing of VesB by two rhomboid proteases in *Vibrio cholerae*

**DOI:** 10.1128/mbio.01270-24

**Published:** 2024-08-13

**Authors:** Cameron S. Roberts, Austin B. Shannon, Konstantin V. Korotkov, Maria Sandkvist

**Affiliations:** 1 Department of Microbiology and Immunology, University of Michigan Medical School, Ann Arbor, Michigan, USA; 2 Department of Molecular and Cellular Biochemistry, University of Kentucky, Lexington, Kentucky, USA; New York University School of Medicine, New York, New York, USA

**Keywords:** proteases, rhomboid, protein secretion, type II secretion system, T2SS, posttranslational modification, *Vibrio cholerae*

## Abstract

Rhomboid proteases are universally conserved and facilitate the proteolysis of peptide bonds within or adjacent to cell membranes. While eukaryotic rhomboid proteases have been demonstrated to harbor unique cellular roles, prokaryotic members have been far less characterized. For the first time, we demonstrate that *Vibrio cholerae* expresses two active rhomboid proteases that cleave a shared substrate at distinct sites, resulting in differential localization of the processed protein. The rhomboid protease rhombosortase (RssP) was previously found to process a novel C-terminal domain called GlyGly-CTERM, as demonstrated by its effect on the extracellular serine protease VesB during its transport through the *V. cholerae* cell envelope. Here, we characterize the substrate specificity of RssP and GlpG, the universally conserved bacterial rhomboid proteases. We show that RssP has distinct cleavage specificity from GlpG, and specific residues within the GlyGly-CTERM of VesB target it to RssP over GlpG, allowing for efficient proteolysis. RssP cleaves VesB within its transmembrane domain, whereas GlpG cleaves outside the membrane in a disordered loop that precedes the GlyGly-CTERM. Cleavage of VesB by RssP initially targets VesB to the bacterial cell surface and, subsequently, to outer membrane vesicles, while GlpG cleavage results in secreted, fully soluble VesB. Collectively, this work builds on the molecular understanding of rhomboid proteolysis and provides the basis for additional rhomboid substrate recognition while also demonstrating a unique role of RssP in the maturation of proteins containing a GlyGly-CTERM.

**IMPORTANCE:**

Despite a great deal of insight into the eukaryotic homologs, bacterial rhomboid proteases have been relatively understudied. Our research aims to understand the function of two rhomboid proteases in *Vibrio cholerae*. This work is significant because it will help us better understand the catalytic mechanism of rhomboid proteases as a whole and assign a specific role to a unique subfamily whose function is to process a subset of effector molecules secreted by *V. cholerae* and other pathogenic bacteria.

## INTRODUCTION

Rhomboid proteases are a family of intramembrane serine proteases that cleave peptide bonds within or adjacent to transmembrane domains (TMDs) (1). GlpG, the rhomboid protease from *Escherichia coli*, has extensively been used to characterize the catalytic mechanism of proteolysis by the rhomboid family (2). A solved crystal structure revealed that GlpG unconventionally uses a catalytic dyad of Ser-His as opposed to the more traditional catalytic triad of Ser-His-Asp used by most serine proteases (3). While a natural substrate has yet to be identified for *E. coli* GlpG (EcGlpG), artificial substrates have been used to probe the substrate specificity around the scission site (P1′P1′) (4). Mutagenesis studies revealed the preference of EcGlpG for small amino acids at the P1 site and bulky residues at P4 and P2′, while there is a preference for positively charged residues at P3 and P2 (1, 4). These results have been corroborated with solved structures of protein-inhibitor complexes (5). A small but growing list of bacterial rhomboid proteases has been characterized with a native substrate, including AraA (rhomboid) and TatA (substrate) from *Providencia stuartii*, YqgP (rhomboid) and MgtE (substrate) from *Bacillus subtilis*, SsGlpG and Rhom7 (rhomboids) and HybA (substrate) from *Shigella sonnei*, and RssP (rhomboid) and VesB (substrate) from *V. cholerae* (6–11).

A bioinformatic study identified a distinct subfamily of rhomboid proteases named rhombosortase (RssP) in Gram-negative bacteria such as *V. cholerae* that maintain the catalytic dyad necessary for proteolysis but lack several features found in GlpG-like rhomboids, such as the cytoplasmic N-terminal domain (Fig. S1) (12). Rhombosortase-like enzymes are widely distributed across the *Vibrio* genus, including pathogens *Vibrio vulnificus* and *Vibrio parahaemolyticus*. Outside of the *Vibrio* genus, rhombosortase-like enzymes were identified in the plant pathogen *Ralstonia solanacearum*, marine animal pathogen *Photobacterium damselae*, the heavy metal remediator *Cupriavidus metallidurans*, opportunistic human pathogen *Aeromonas hydrophilla*, and notable nosocomial pathogen *Acinetobacter baumannii* (13–17). *V. cholerae* also encodes a homolog of EcGlpG. Excluding the N-terminal domain, RssP shares 32% and 23% identity with GlpG and EcGlpG, respectively, while GlpG and EcGlpG share 43% identity over their entire sequence. Rhombosortase-like enzymes co-distribute with proteins that contain a C-terminal tripartite extension consisting of a glycine-rich region, TMD, and positively charged terminus collectively called the GlyGly-CTERM (12). For example, *V. cholerae* encodes RssP and six proteins with a GlyGly-CTERM, including three serine proteases VesA, VesB, and VesC, the DNase Xds, a putative metalloprotease VCA0065, and VC1485, a protein of unknown function. Subsequent biochemical characterization validated the GlyGly-CTERM protein VesB as a bona fide substrate of RssP in *V. cholerae* (10). The serine protease VesB undergoes a number of processing steps during its maturation, including three proteolytic events. First, it is transported by the Sec pathway to the periplasm with the aid of its N-terminal signal peptide, which is eventually cleaved off by signal peptidase (event 1). During translocation, the GlyGly-CTERM anchors VesB to the inner membrane. Cleavage of the GlyGly-CTERM by RssP (event 2) coincides with a posttranslational modification event by a yet-to-be determined mechanism, which allows for cell surface localization after transport through the outer membrane by the type 2 secretion system (T2SS) (10). While previous liquid chromatography-tandem mass spectrometry of purified VesB suggested that the newly generated C-terminus is modified with a glycerophosphoethanolamine moiety following cleavage by RssP, the mechanism of this modification has not been described to date (10). At the surface, VesB undergoes activation through auto-catalytic removal of its N-terminal propeptide (event 3) (10). Subsequent release of a fraction of surface localized VesB to the extracellular space may occur via outer membrane blebbing and/or proteolytic cleavage (10). In addition, VesB can also be cleaved by GlpG, albeit with decreased efficiency, which is most evident in the absence of RssP (10). However, the site and consequence of GlpG cleavage have not been fully investigated. Here, we examine the molecular mechanisms of the two distinct rhomboid proteases using their shared native substrate and demonstrate unique substrate recognition and specificity of VesB by RssP and GlpG.

## RESULTS

### RssP is required for the maturation of active VesB and surface localization

**Fig 1:**
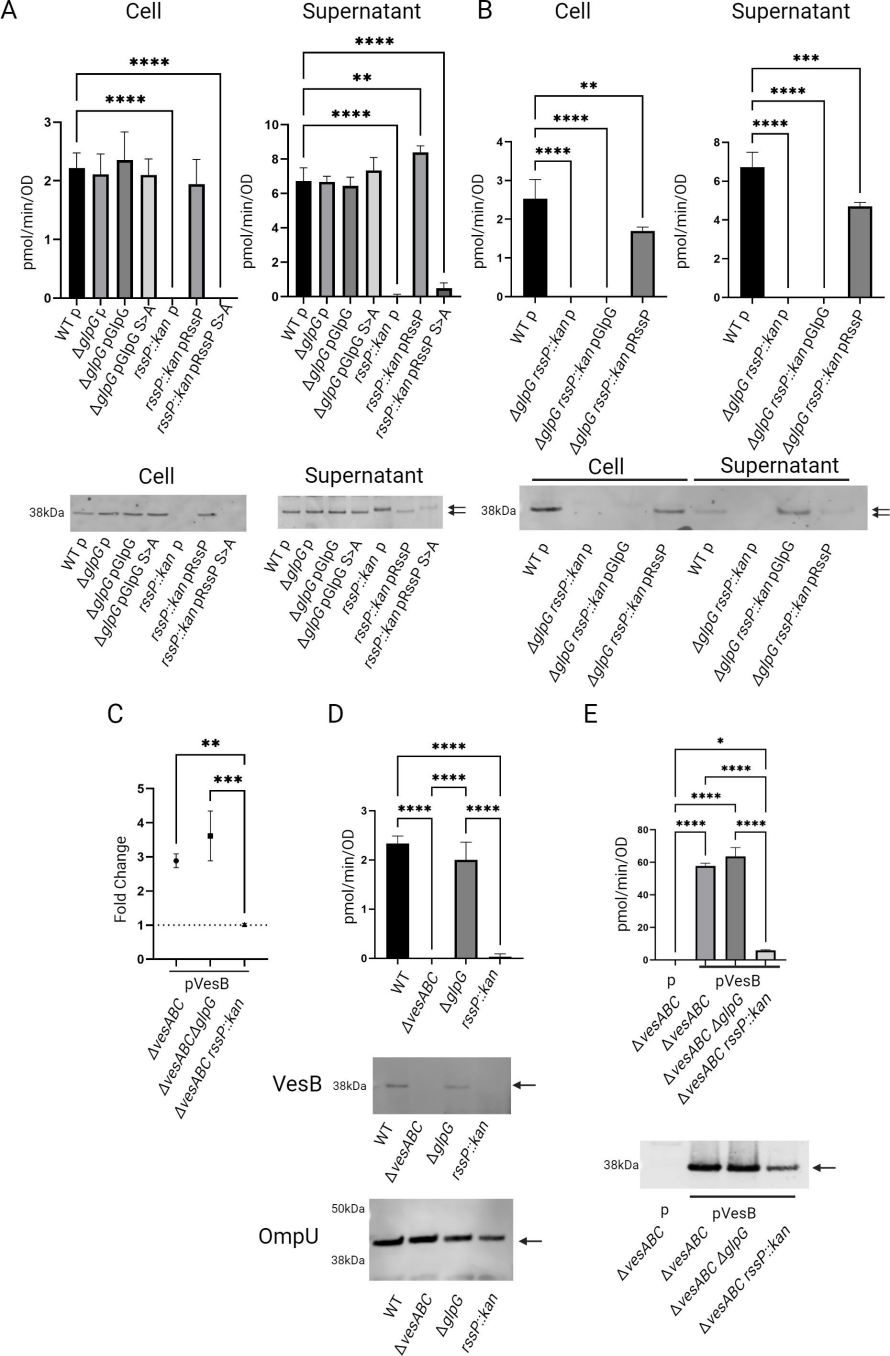
RssP uniquely targets active VesB to the cell surface and outer membrane vesicles. (A) Cell and supernatant fractions from overnight cultures of WT and indicated rhomboid mutant strains with empty vector (p) or plasmid encoding active or inactive (S > A) rhomboid proteases RssP or GlpG were assessed for natively expressed serine protease activity assayed against the fluorogenic peptide Boc-Gln-Ala-Arg-AMC. Data represent mean ± SD of *n* = 3 experiments. The same samples were also run on sodium dodecyl sulfate-polyacrylamide gel electrophoresis (SDS-PAGE), transferred to a nitrocellulose membrane, and blotted with anti-VesB antibodies. Arrows indicate active VesB with its propeptide removed (single arrow or bottom) and inactive VesB (top). (B) Cell and supernatant fractions from overnight cultures of WT and the double rhomboid mutant strain with empty vector (p) or plasmid encoding active rhomboid proteases RssP or GlpG were assessed for natively expressed serine protease activity assayed against the fluorogenic peptide Boc-Gln-Ala-Arg-AMC. Data represent mean ± SD of *n* = 3 experiments. The same samples were also run on SDS-PAGE, transferred to a nitrocellulose membrane, and blotted with anti-VesB antibodies. Arrows indicate active VesB with its propeptide removed (single arrow or bottom) and inactive VesB (top). (C) VesB was ectopically overexpressed (50 µM) in the Δ*vesABC* mutant strain and in the same mutant strain with either *glpG* deleted (Δ*glpG*) or *rssP* disrupted (*rssP::kan*). Cell fractions were isolated after growth in M9 media supplemented with casamino acids and glucose, and the surface exposed VesB was probed by incubating with anti-VesB followed by incubation with goat anti-rabbit IgG coupled with Alexa Fluor 488 and scanning for fluorescence emission intensity in 96-well format. Fold change was determined by dividing the fluorescence intensity produced by VesB-expressing strains over control strains containing empty vector (mean ± SD of *n* = 3). (D) Filtered supernatants from overnight cultures of WT, Δ*vesABC,* Δ*glpG,* or *rssP::kan* mutant strains were subjected to high-speed centrifugation to separate crude outer membrane vesicles from the cleared supernatant. The pelleted fraction was resuspended in Luria-Bertani and subjected to serine protease activity determination and Western blotting as described in panel A (mean ± SD of *n* = 3). For the blot, 10 times the concentration compared to fractions assessed for activity was analyzed. As a control, samples were also blotted for OmpU (lower). (E) VesB was ectopically overexpressed (50 µM isopropyl β-d-1-thiogalactopyranoside [IPTG]) in the Δ*vesABC* mutant strain with and without *glpG* deleted (Δ*glpG*) or *rssP* disrupted (*rssP*::kan). Culture supernatants were assayed for activity using the same fluorogenic peptide as in panel A (mean ± SD of *n* = 3). Supernatants were also probed for VesB using anti-VesB antibodies. Arrow indicates active VesB. *P < 0.05, **P < 0.01, ***P < 0.001, and ****P < 0.0001 by one-way ANOVA analysis with Dunnett’s multiple comparisons test (A and B) and one-way ANOVA analysis with Tukey’s multiple comparisons test (**C–E**). Representative Western blots are shown from at least two blots performed on biological replicas.

To probe the dependence of VesB localization on rhomboid cleavage, cell and culture supernatants from *rssP* and *glpG* mutant strains expressing either wild-type (WT) or catalytically inactive (Ser > Ala) RssP or GlpG were analyzed using a fluorogenic peptide specific to the enzymatic activity of VesB (Fig. 1A). The activity of chromosomally expressed VesB was not detected in the culture supernatant or whole cell fractions in the absence of RssP, while ectopically expressed RssP but not RssP-S102A restored the activity in both fractions. Subsequent Western blotting confirmed an absence of VesB in the cell fraction lacking active RssP. Interestingly, unlike the cell fraction, VesB antigen was detected in the culture supernatant but with a slightly elevated molecular weight, indicating an inactive VesB that failed to remove its propeptide. In contrast, deletion of *glpG* had no effect on the localization or activity of VesB (Fig. 1A). In a double rhomboid (*rssP::*kan*,* ∆*glpG*) deletion strain, we could not detect VesB in any fraction by activity or Western blot. We previously demonstrated that in the absence of both rhomboid proteases, overexpression of a VesB construct was required to detect VesB at a low level in the cell fraction, consistent with VesB remaining trapped in the inner membrane and subject to degradation in this strain (10). Here, while complementation with RssP restored WT-level activity in cell and supernatant fractions, GlpG complementation resulted in the release of VesB to the culture supernatant similar to the *rssP*::kan strain (Fig. 1B).

Consistent with the absence of VesB in the cell fraction in the *rssP::*kan mutant, we confirmed that VesB was not retained on the cell surface by incubating intact cells with antibodies raised against VesB followed by incubation with fluorescently labeled secondary antibodies and fluorometry, while VesB was detected on the surface at WT levels in the *glpG* deletion strain (Fig. 1C). These results are consistent with previous findings indicating that GlpG can cleave VesB in the absence of RssP and demonstrate that RssP, in addition to a functioning T2SS, is required for the surface retention of VesB (10). Since RssP is necessary for the surface localization of VesB, we hypothesized that it is also required for release through outer membrane vesicles (OMVs). VesB had previously been found in the pelletable fraction of culture supernatants also containing OmpU (10). To test RssP dependence of OMV localization, culture supernatants from WT and indicated rhomboid mutants were pelleted to isolate crude OMVs, which were assessed for serine protease activity (Fig. 1D). We found that serine protease activity reported with the fluorogenic peptide specific to VesB (and to a lesser extent VesC [18]) is associated with OMVs from WT and *glpG* mutant strains, but not OMVs of the mutant with *rssP* disrupted. The magnitude of the detected protease activity is approximately one-third of that detected in total supernatants in Fig. 1A. In the corresponding Western blot, VesB is observed in OMVs from WT and the *glpG* mutant strain. In contrast, the *rssP*::kan strain did not have detectable serine protease activity or an apparent VesB antigen in the OMV fraction. As a control, pelleted supernatants were blotted against OmpU, a major component of OMVs (19).

Since, in the absence of RssP, GlpG can release VesB to the culture supernatant, we were curious if this species was still functionally competent (10). To this end, we ectopically overexpressed VesB with 50 µM IPTG induction in the WT and different rhomboid mutant strains (Fig. 1E). Under these conditions, low VesB activity was observed in the culture supernatant of the *rssP*::kan strain. Also, consistent with VesB activity, the corresponding Western blot showed a band for VesB of a similar molecular weight as VesB expressed in the WT or Δ*glpG* strains. This finding suggests that surface localization of VesB allows for more efficient activation (i.e., removal of its propeptide) compared to simply releasing the protease to the culture supernatant, which is mediated by GlpG when RssP is absent. To address the molecular mechanisms of the differential localization of VesB cleaved by RssP and GlpG, we investigated the cleavage specificity of both GlpG and RssP.

### RssP cleaves within the TMD of the VesB GlyGly-CTERM domain

**Fig 2:**
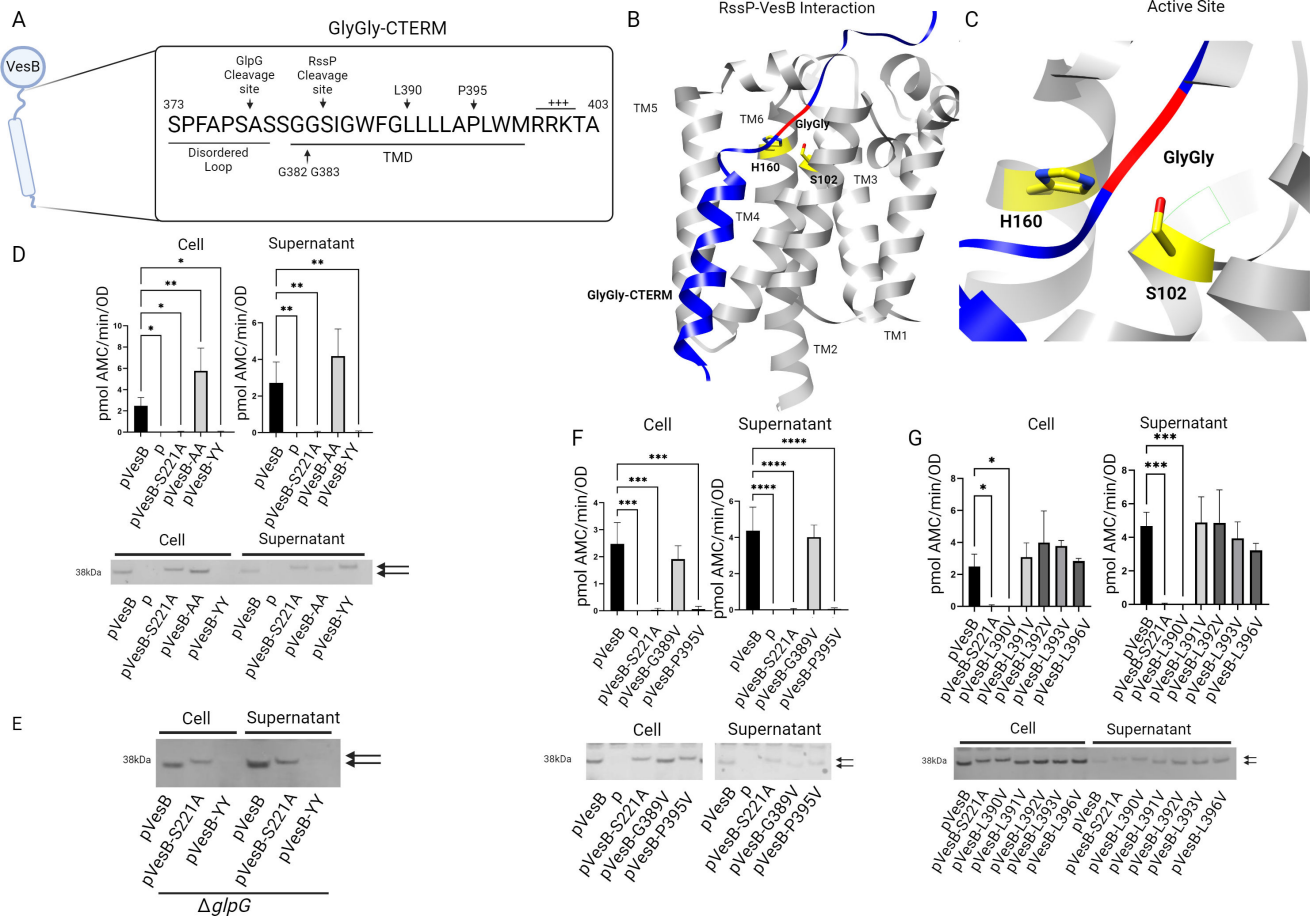
RssP cleaves VesB after glycine 383 and requires both a helix kinking residue and conserved leucine residue with the TMD of VesB. (A) Schematic of VesB is shown with a focus on the GlyGly-CTERM domain. GlpG and RssP cleavage sites are indicated as determined in this study. The disordered loop, transmembrane domain, and positively charged tail are labeled along with important amino acids for RssP-mediated cleavage. (B) A predicted interaction of RssP (gray) and VesB (blue) was generated using AlphaFold2. RssP and the GlyGly-CTERM of VesB are shown, including the active site residues (yellow) and TMDs of RssP and the GlyGly signal (red) of VesB. (C) A close-up of the predicted structure in panel B shows the active site of RssP with VesB threaded through. Predicted alignment error is shown in Fig. S3A. (D) Overnight cultures of the mutant strain Δ*vesABC* containing empty vector (p) or plasmids encoding WT VesB or VesB with di-alanine (AA) and di-tyrosine (YY) substitutions of the di-glycine motif were separated into cell and supernatant fractions and assessed for serine protease activity using the fluorogenic peptide substrate Boc-Gln-Ala-Arg-AMC. Data represent mean ± SD of *n* = 3 experiments. Fractions were also run on sodium dodecyl sulfate-polyacrylamide gel electrophoresis (SDS-PAGE), transferred to a nitrocellulose membrane, and blotted with anti-VesB antibodies. (E) Cultures of the mutant strain Δ*vesABC*Δ*glpG* containing empty vector (p) or plasmids encoding VesB constructs were separated into cell and supernatant fractions and run on SDS-PAGE, transferred to a nitrocellulose membrane, and blotted with anti-VesB antibodies. (F) VesB with G389V and P395 substitutions were compared to WT VesB and similarly processed as in panel D (mean ± SD of *n* = 3). (G) VesB with valine substitutions in the leucine-rich region was compared to WT VesB and processed as in panel D (mean ± SD of *n* = 3). Arrows indicate active (bottom) and inactive (top) VesB. *P < 0.05, **P < 0.01, ***P < 0.001, and ****P < 0.0001 by one-way ANOVA with Dunnett’s multiple comparisons test. Representative Western blots are shown from at least two blots performed on biological replicas.

Mass spectrometry peptide fragmentation data from purified VesB suggested RssP cleaves VesB at position G383 (Fig. 2A) (10). To validate and further map this cleavage site of VesB, a predicted protein complex was generated representing the association of VesB and RssP using AlphaFold2 (20). Extraordinarily, several models showed the GlyGly-CTERM of VesB threaded through the active site and TMD helices of RssP (Fig. 2B and C). As seen with structures of other rhomboids in complex with inhibitors, the TMD of VesB is predicted to interact with TMs 2 and 5 of RssP (5, 21). The active site residues of RssP, S102, and H160 on TMs 4 and 6, respectively, are brought into proximity and contact with G382 and G383 of the TMD of VesB, where there appears to be local unwinding of the TMD helix around the glycine residues. In additional predicted structures, the TMD of VesB interacts with TMD 2 and 5 of RssP, but the L5 cap of RssP seems to block VesB entry into the RssP catalytic site (Fig. S2A and B). These two predicted structures may represent the scission and docking complexes, respectively, where RssP first recognizes VesB in the docking complex (Fig. S2A) followed by proteolysis facilitated with the scission complex (Fig. 2B). It should be noted that the predicted interactions of the TMD of VesB with RssP have low pAE values, while other domains of VesB have high pAE values (Fig. S3A and B). Therefore, the predicted interactions are only used as the hypotheses driving experimental design along with peptide mapping and sequence conservation to initiate mutagenesis studies.

To test if the double glycine motif (G382–G383) within the TMD of the GlyGly-CTERM is required for RssP cleavage, conservative (alanine) and non-conservative (tyrosine) VesB double mutants were generated and tested for VesB activity when ectopically expressed. VesB-AA displayed WT VesB-level activity in the supernatant fraction and enhanced activity in the cell fraction, indicating that alanine residues at positions 382 and 383 are permissible to RssP cleavage (Fig. 2D). On the other hand, VesB-YY did not have any measurable activity in either the cell or supernatant fraction comparable to empty vector (p) or catalytically inactive VesB-S221A. When blotted with VesB antibodies, VesB-YY was detected only in the supernatant at a slightly higher molecular weight, which indicates inactive VesB. This pattern strongly implied that VesB-YY was only cleaved by GlpG with perhaps more efficiency than WT VesB. Indeed, in a Δ*glpG* background, the apparent VesB-YY band in the supernatant fraction was lost (Fig. 2E). We speculate that uncleaved VesB-YY accumulated in the inner membrane and was subsequently degraded by intracellular proteases similar to WT VesB when expressed in a mutant deficient in both RssP and GlpG (Fig. 1B). Similar to WT VesB expressed in a *rssP::*kan mutant strain (Fig. 1E; Fig. S4A), VesB-YY released to the culture supernatant could be activated in the Δ*vesABC* mutant when overexpressed with IPTG (Fig. S4B).

### RssP cleavage of VesB requires conserved leucine and proline residues

Since RssP cleaves VesB within the TMD domain and helix-breaking residues are known to be important for cleavage when rhomboid proteases cleave their target substrate within its TMD (1), the conserved helix-breaking residues G389 and P395 in the GlyGly-CTERM were also subjected to mutagenesis. While mutating G389 produced no observable change in phenotype as compared to WT VesB, abolishing the helix-breaking residue P395 by mutating it to valine resulted in inactive VesB with higher molecular mass associated with the cell and supernatant fractions (Fig. 2F). This blotting pattern is consistent with partial cleavage of VesB-P395V by GlpG and release to the culture supernatant. The cell-associated VesB-P395V may indicate an intracellular location since this VesB species is inactive. When expressed in a Δ*glpG* strain this construct was only found in the cell fraction (Fig. S5).

Previously, it was highlighted that there is an enrichment of leucine residues (34%) in GlyGly-CTERM TMDs compared to all inner membrane-spanning proteins (17%) (12). Therefore, site-specific mutations were also generated at each position with a leucine residue in the GlyGly-CTERM of VesB. Valine substitution at position 390 resulted in inactive VesB associated with cell and supernatant fractions, similar to the P395V and S221A substitutions (Fig. 2G). Expression in a Δ*glpG* mutant strain revealed that GlpG primarily cleaves this construct (Fig. S5). However, the other Leu substitutions had no measurable effect on VesB. These results indicate that L390 is required for RssP recognition of VesB, while all other Leu residues are dispensable for processing by RssP and subsequent auto-activation of VesB. Even a strain with all leucine residues mutated to valine behaved similarly to WT VesB (Fig. S6A and B).

### The highly conserved “SGGS” of the VesB motif is sensitive to amino acid substitutions

**Fig 3:**
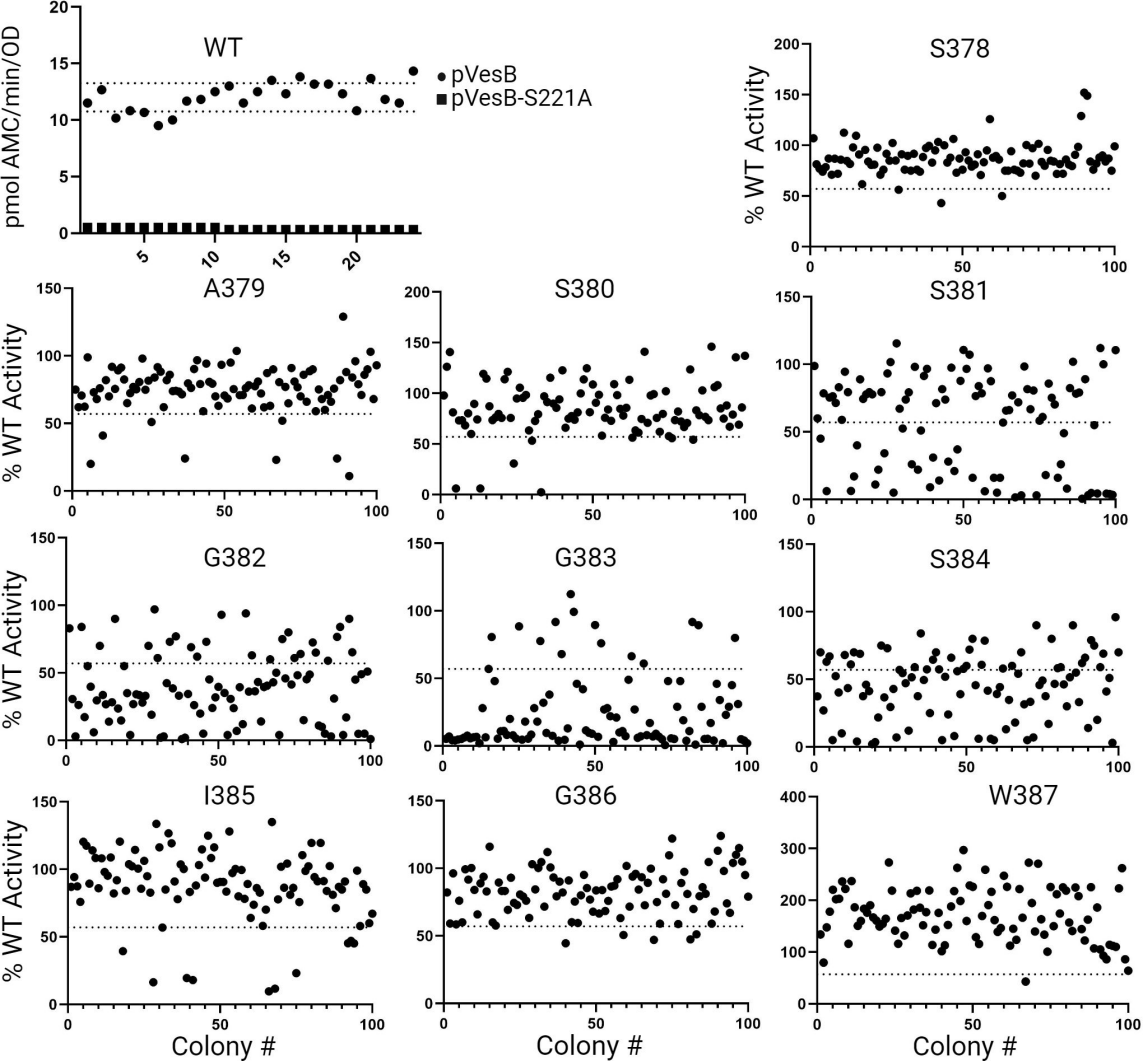
The highly conserved “SGGS” motif within the GlyGly-CTERM is sensitive to substitutions. Cultures from individual colonies of Δ*vesABC* ectopically expressing either WT VesB or VesB variants were grown overnight in Luria-Bertani without induction in a 96-well format. Wells were directly assessed for serine protease activity using the fluorogenic peptide substrate Boc-Gln-Ala-Arg-AMC without separating cells and culture supernatants. First, 24 colonies each of Δ*vesABC* ectopically expressing either VesB or the active site mutant (VesB-S221A) were tested (WT). Each data point represents the activity from an overnight culture of an individual colony. Mean (12.0 pmol AMC/min/OD) ± SD (1.25 pmol/min/OD) for the 24 cultures expressing WT VesB is shown and indicated with dotted lines. Next, a library of VesB plasmids was generated with degenerate primers “NNK” at the indicated amino acid position before conjugation into the Δ*vesABC* mutant strains where cultures of individual colonies were grown and processed for activity (indicated mutant). Each point represents a culture from an individual colony, and the activity is represented as a percentage of the average activity of WT VesB expressed in the Δ*vesABC* strain. The dotted line indicates four standard deviations below the normalized 100% WT VesB activity. For each amino acid position, cultures of 100 colonies were analyzed.

To further define the RssP cleavage site of VesB, site-saturation mutagenesis was performed on residues S378–W387. The primers containing a degenerative NNK codon at the chosen amino acid positions were used to create a library of VesB mutants that were ectopically expressed and screened for VesB activity in 96-well plate format. As proof of concept and to determine potential well-to-well variation, *V. cholerae* expressing WT VesB and catalytically inactive VesB were grown in 96-well plates and assayed for activity (Fig. 3). Then, an initial 100 colonies were selected to test for activity at each position subjected to mutagenesis, and the activity of individual colonies in relation to WT VesB at each amino acid position is shown in Fig. 3. These results show that positions S381, G382, G383, and S384 were sensitive to mutagenesis generating mostly inactive VesB variants, while positions S378, A379, S380, I385, G386, and W387 were not. It is not clear at this moment why W387 substitutions resulted in increased activity compared to WT VesB. At each of these sensitive positions, an additional 100 colonies were screened, and their identity was determined using Sanger sequencing, where the screen could identify 13–18 amino acids that resulted in significantly reduced VesB activity below four SD of WT VesB activity. The amino acids at each of the positions that were not initially identified in this screen were generated using site-specific primers, and the activity of each amino acid substitution at each position is shown in Table 1. As the most sensitive site, only alanine, cysteine, and glutamate were acceptable for comparative WT VesB activity at position 383. Alanine is present at this position in some rhombosortase targets (12). The reason why the glutamate substitution is acceptable has yet to be understood; however, we speculate that cysteine is permissible due to its similar size to glycine. Similarly, cysteine substitution at the corresponding position in TatA resulted in WT levels of AraA-mediated TatA cleavage, but not glutamate substitution (1). Position 382 was also sensitive to mutagenesis but could accommodate additional bulkier and charged residues such as valine, isoleucine, lysine, tryptophan, histidine, and phenylalanine, albeit with reduced activity for some. Position 384 could accommodate additional residues, asparagine and methionine, similar to position 382, while position 381 shared a similar profile but could not accommodate the negatively charged glutamate. Of all the bioinformatically identified GlyGly-CTERM proteins, glycine residues at the equivalent 382 and 383 positions are most conserved, while the serine residues at positions 381 and 384 are less conserved (12). These results, along with previously published peptide mapping of purified VesB samples, strongly point to RssP cleavage of VesB at G383 and show the “SGGS” motif is critical for VesB GlyGly-CTERM processing and subsequent VesB activation (10). Compared to *E. coli* GlpG, RssP conserves a preference for small amino acids at P1 but does not have an amino acid preference at P4 or P2′. Instead, the P3 to P1′ positions appear to be critical for substrate cleavage where small hydrophobic amino acids are generally preferred, and P2 and P1 represent the most critical positions.

**TABLE 1:** The substrate specificity of VesB cleavage by RssP at the “SGGS” motif is revealed through site-saturation mutagenesis^*b*^

Amino acid substitution	S381 (% activity)	G382 (% activity)	G383 (% activity)	S384 (% activity)
A	3	116^*a*^	153^*a*^	>1
C	2	140^*a*^	110^*a*^	140^*a*^
D	3	2	>1	87^*a*^
E	3	101^*a*^	60	55^*a*^
F	4	42^*a*^	>1	6
G	>1	100 (WT)	100 (WT)	>1
H	85^*a*^	48	>1	50
I	180^*a*^	86^*a*^	5	121^*a*^
K	123^*a*^	49	>1	3
L	5	4	2	11
M	102^*a*^	2.5	>1	94^*a*^
N	2.5	>1	>1	147^*a*^
P	36	4	>1	14
Q	8	4	>1	38
R	2	4	>1	2
S	100 (WT)	5	6	100 (WT)
T	8	4	>1	>1
V	79^*a*^	119^*a*^	>1	63
W	180^*a*^	52	>1	>1
Y	79^*a*^	>1	4	>1

^*a*^ Residues not detected in the site-saturating screen and were therefore individually constructed using site-specific primers.

^*b*^ WT indicates residue present in WT VesB.

### GlpG cleaves VesB within the disordered loop preceding the GlyGly-CTERM

**Fig 4:**
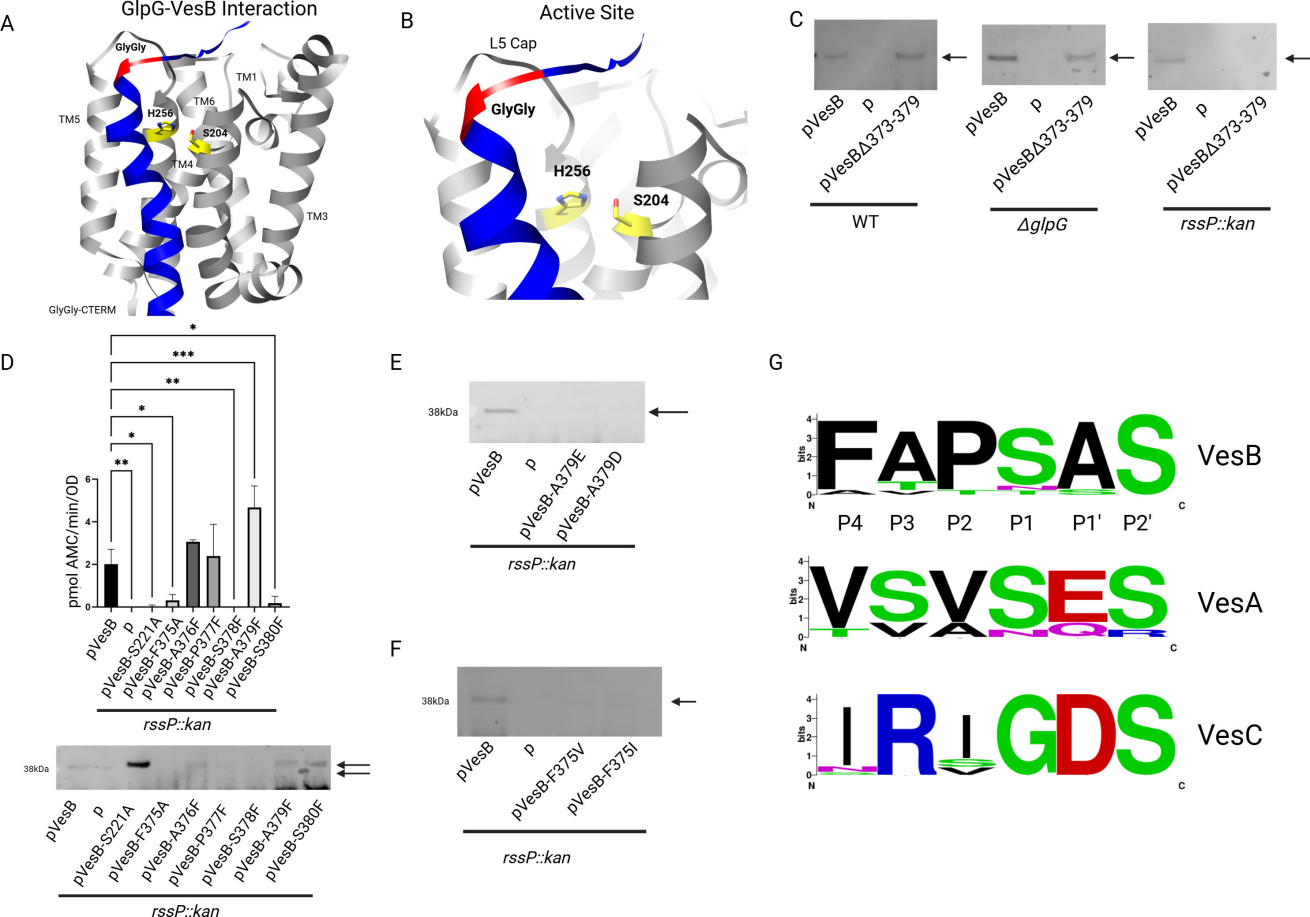
GlpG cleaves VesB within the disordered loop of the GlyGly-CTERM when RssP is absent. (A) Predicted AlphaFold2 interaction of GlpG (gray) with VesB (blue) (upper). The TMDs and catalytic residues (yellow) of GlpG are indicated as well as the GlyGly signal of VesB (red). (B) A close-up of panel A showing the GlpG active site and VesB disordered loop is shown (lower). Predicted alignment error is shown in Fig. S3C. (C) Supernatants from cultures of either Δ*vesABC*, Δ*vesABCΔglpG*, and Δ*vesABC rssP*::kan strains containing pVesB or pVesBΔ373–379, which codes for a VesB construct with residues F375-A379 deleted, were run on sodium dodecyl sulfate-polyacrylamide gel electrophoresis (SDS-PAGE), transferred to a nitrocellulose membrane, and blotted with anti-VesB antibodies. The arrow indicates active VesB. (D) Supernatants from cultures induced with 50 µM IPTG of the Δ*vesABC rssP*::kan mutant strain containing either empty vector, pVesB, or indicated plasmids with VesB substitutions were assessed for serine protease activity using the fluorogenic peptide Boc-Gln-Ala-Arg-AMC control (mean ± SD of *n* = 3). *P < 0.05, **P < 0.005, and ***P < 0.0005 by one-way ANOVA with Dunnett’s multiple comparisons test. Fractions were also run on SDS-PAGE, transferred to a nitrocellulose membrane, and blotted with anti-VesB antibodies. Arrows indicate active (bottom) and inactive (top) VesB. (E) Supernatants from cultures induced with 50 µM IPTG of the Δ*vesABC rssP*::kan mutant containing empty vector, pVesB, and plasmids with VesB A379E or A379D substitutions were probed by Western blotting. (F) Samples are similarly prepared as in panel D but VesB F375A and F375I substitutions are shown. The arrow indicates active VesB. Representative Western blots are shown from at least two blots performed on biological samples. (G) The GlyGly-CTERM from VesA, B, and C were aligned to identify possible GlpG-cleavage sites in VesA and VesC. Each GlyGly-CTERM was blasted using the NCBI clustered database where all sequences were extracted, and a sequence logo of the desired positions was generated. VesB P4-P2′ cleavage positions of GlpG are listed. The equivalent residues are shown for VesA and VesC.

To identify the GlpG cleavage site of VesB, a model of VesB and GlpG complex was generated using AlphaFold2. VesB was predicted to interact with GlpG through TM2 and TM5 of GlpG (Fig. 4A). Of the top-ranked models, the predicted structures closely resembled a docking complex akin to the RssP-VesB prediction in Fig. S2A. In the GlpG-VesB structure, the L5 cap, which gated the RssP-VesB docked structure, is not as ordered as in the RssP-VesB structure and potentially the disordered loop of VesB would have access to the catalytic residues of GlpG (Fig. 4B). We also note that the local unwinding seen around the glycine region in the RssP-VesB structure is not predicted in the GlpG-VesB structure.

Since the predicted structure agreed with our results indicating GlpG cleaves VesB distal to the glycine-rich region and outside the TMD, the disordered loop in VesB (S373–A379) was specifically deleted, keeping the rest of the protein intact, including the rest of the GlyGly-CTERM (VesBΔ373–379). In the WT background, equal amounts of ectopically expressed WT VesB and VesBΔ373–379 were detected in culture supernatants by Western blot (Fig. 4C). We speculate that this is because RssP is still able to cleave VesBΔ373–379. Indeed, in the *rssP::*kan mutant, the corresponding Western blot showed no VesBΔ373–379 released to the culture supernatant, unlike the *ΔglpG* strain where both WT and VesBΔ373–379 were detected in the culture supernatant. These results indicate that VesBΔ373–379 is no longer cleaved by GlpG and suggest that GlpG primarily cleaves VesB within the disordered loop.

To further characterize the GlpG cleavage site, phenylalanine scanning mutagenesis was performed on the disordered loop of VesB, and the resulting constructs were expressed in the *rssP::*kan mutant strain and probed for activity and location. Phenylalanine was chosen over other residues, such as alanine, because rhomboid proteases, like elastase, prefer small residues at the P1 and P1′ positions (8, 21, 22). Since the identity of position 375 is already phenylalanine, we mutated this residue to alanine. Of the positions tested, VesB with F375A, S377F, and S380F substitutions had low activity in the culture supernatant in the absence of RssP, while VesB-S378F had no measurable activity (Fig. 4D). Western blotting detected bands for WT VesB and VesB with the A376F, A379F, and S380F substitutions, while no bands were observed for VesB with either F375A or S378F. We note that the stability of VesB-S221A is slightly increased compared to both WT VesB and the mutant constructs in the *rssP*::kan mutant and speculate that active VesB may induce some stress in the cell, possibly extracytoplasmic stress, resulting in some degradation of VesB. Extracytoplasmic stress may also explain why the *rssP*::kan mutant is sensitive to polymyxin B (23). The stress may be further exacerbated in the rhomboid double mutant. If we consider S378 as P1 and F375 as P4 for GlpG, these positions were also identified as being the most sensitive to mutagenesis for the rhomboid protease AraA from *P. stuartii*. Small amino acids are preferred at position P1, while there is a preference for bulky amino acids at P4 (11). Like preferences found for AraA, GlpG cleavage of VesB did not depend on the amino acid identity at positions P2, P3, and P1′. The proposed cleavage site is consistent with the deletion of the disordered loop, the known substrate specificity of GlpG-like rhomboids, and the irrelevance to the helix-breaking residue P395 within the TMD of the GlyGly-CTERM. None of the mutations investigated in the disordered loop had a negative effect on the subsequent activation of VesB in a WT background, indicating that cleavage by RssP was intact (Fig. S7). The notion that GlpG cleaves at position S378 is also supported by previous mass spectrometry analysis of VesB purified from culture supernatants of a WT *V. cholerae* overexpressing VesB where peptides were identified ending at position 378 (10).

Mutagenesis studies of TatA, the substrate of AraA, indicate that a glutamate is accepted at P1′, but not an aspartate, presumably due to the increase in side-chain length (11). A379, the proposed P1′ residue in VesB, was mutated to both glutamate and aspartate, and the activities of the ectopically expressed mutant VesB constructs in the *vesABC rssP*::kan mutant were determined (Fig. 4E). In contrast to AraA cleavage of TatA, both the VesB-A379E and VesB-A379D constructs had significantly reduced activity in culture supernatants, and a band was not detected in the corresponding Western blot fractions. In addition, when VesB-F375 was substituted with either valine or isoleucine, loss of activity for these mutant VesB species, compared to WT VesB, showed that phenylalanine is critical at this position for GlpG cleavage (Fig. 4F). TatA has an isoleucine at this position but also tolerates valine and phenylalanine substitutions. When we examined the C-terminal sequences of the six identified GlyGly-CTERM proteins from *V. cholerae* strain N16961, we found that the GlpG-cleavage site is only conserved in VesB. Across all *Vibrio* species with available sequences in NCBI, the GlpG cleavage site in VesB homologs is highly conserved (Fig. 4G). VesA and VesC homologs are also highly conserved across *Vibrio* species but contain unfavorable residues at the equivalent P4 and P1′ positions that are predicted to block GlpG cleavage. While yet to be directly confirmed, this might indicate that GlpG has a unique role in the extracellular release of VesB but not the closely related VesA and VesC.

### GlpG preferentially cleaves a VesB chimera containing the TMD of a GlpG substrate from *S. sonnei*

**Fig 5:**
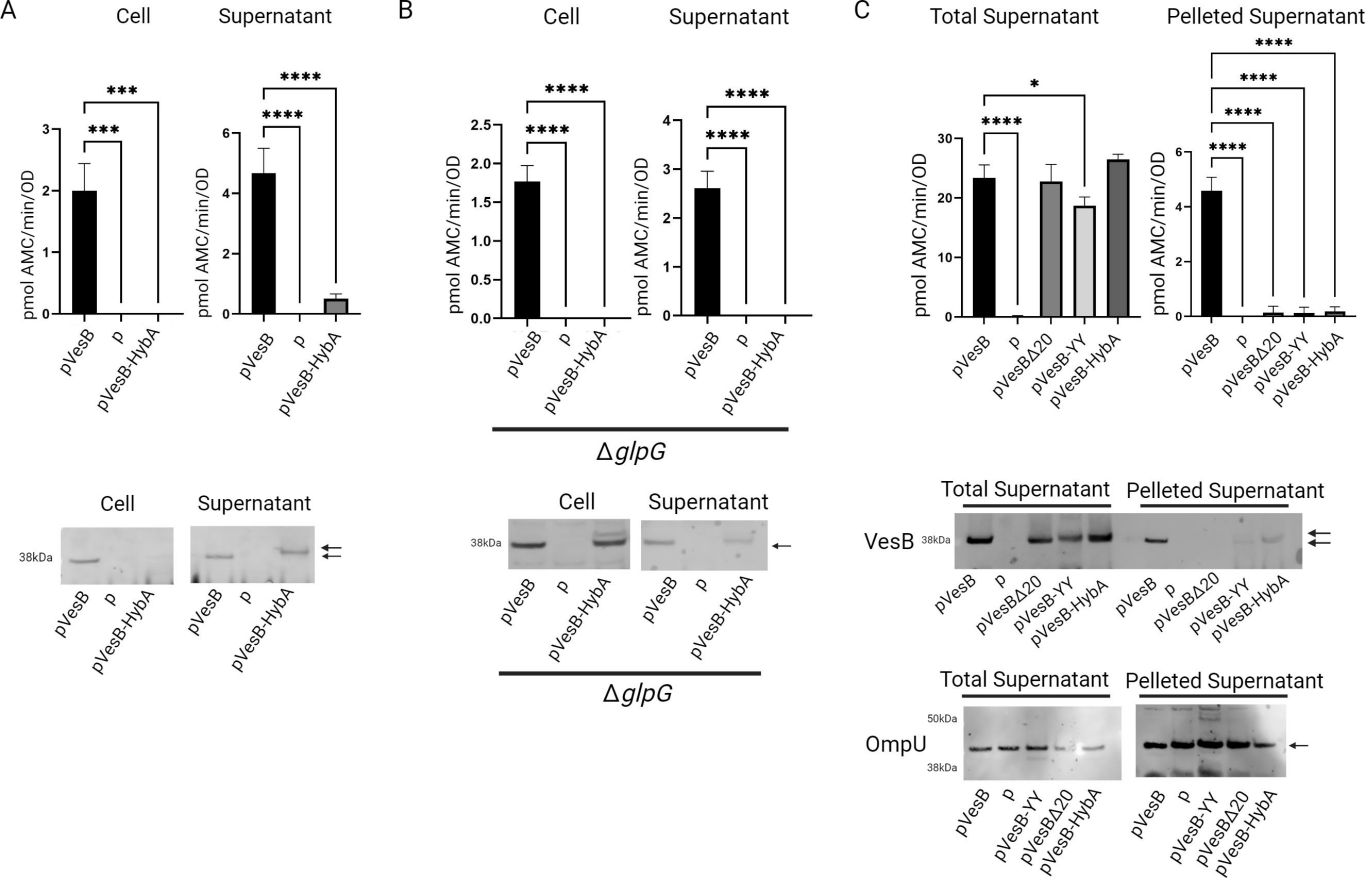
The TMD of VesB drives substrate recognition and protein targeting by the rhomboid proteases in *V. cholerae*. Culture supernatants of either Δ*vesABC* (**A**) or Δ*vesABCΔglpG* (**B**) strains containing pVesB or pVesB-HybA, which codes for a VesB construct with the TMD residues of VesB swapped for those of the TMD of HybA from *S. sonnei*, were assessed for serine protease activity using the fluorogenic peptide Boc-Gln-Ala-Arg-AMC. Data represent mean ± SD of *n* = 3 experiments. Fractions were also run on sodium dodecyl sulfate-polyacrylamide gel electrophoresis, transferred to a nitrocellulose membrane, and blotted with anti-VesB antibodies. (C) Supernatants from cultures induced with 10 µM IPTG of Δ*vesABC* mutant strain containing empty vector (p), pVesB, pVesB-YY, pVesBΔ20, or pVesB-HybA were sterile filtered and subjected to high-speed centrifugation to separate crude outer membrane vesicles from the cleared supernatant. The pelleted fractions were resuspended in Luria-Bertani and subjected to serine protease activity and Western blotting as described in panel A (mean ± SD of *n* = 3). Pelleted fractions were loaded at 10 times the concentration of total supernatant. As a control, samples were also blotted for OmpU (bottom). *P < 0.05, ***P < 0.0001, and ****P < 0.0001 by one-way ANOVA with Dunnett correction comparison to WT. Representative Western blots are shown from at least two blots performed on biological replicas.

To see if RssP can recognize the TMD of a known GlpG substrate, we created a chimeric protein consisting of VesB and the portion of the TMD C-terminal to S384 swapped for the TMD of *S. sonnei* HybA (GenBank UXF90189). The TMD from HybA (N′-TLYK**G**MMLPLAVLA-C′) was determined bioinformatically using the DeepTMMHM server, where the native cleavage site was previously identified as occurring after residue G296 in HybA, highlighted in bold (9, 24). The positively charged tail of VesB was kept intact to properly orient VesB in the inner membrane. HybA was chosen over the two other identified bacterial rhomboid substrates, TatA and MgtE, because, like VesB, it is a C-terminally anchored single-pass inner membrane protein. This chimera was assessed for localization and activity in *V. cholerae* (Fig. 5A). Strikingly, VesB-HybA displayed a similar localization to GlpG-cleaved WT VesB as it was only detected in supernatant fractions with Western blotting. VesB-HybA had significantly reduced activity in the culture supernatants compared to WT VesB even though RssP was still present, which we speculate is because this construct is primarily cleaved by GlpG. Indeed, when the same experiment was performed in a Δ*vesABC*Δ*glpG* mutant, there was a loss of activity in the supernatant fraction for the VesB-HybA construct, and the majority was now detected by Western blot in the cell fraction (Fig. 5B). A small fraction of the VesB-HybA construct was detected in the supernatant in this strain, suggesting that RssP does cleave this construct, but with decreased efficiency in the absence of GlpG. To determine if GlpG cleaves at the identified *S. sonnei* GlpG cleavage site in the HybA sequence, two separate substitutions of this site, glycine to either valine or arginine, were made that prevent cleavage by *S. sonnei* GlpG. Both constructs, like the original VesB-HybA chimera, were similarly targeted to the supernatant, indicating GlpG cleaved at its native site in VesB rather than in the HybA sequence of this construct (Fig. S8A). Cell-associated VesB-HybA found in the Δ*glpG* mutant was also confirmed to be contained within the cell (Fig. S8B).

### Genetic deletion of part of the GlyGly-CTERM of VesB results in a soluble construct that behaves similarly to a GlpG-cleaved product

To investigate the effect of genetically removing 20 residues of the VesB C-terminal of the double glycine, we generated a VesB construct, VesBΔ20, that lacks the same residues that RssP proteolytically removes. Activity assay and Western blotting showed that VesBΔ20 was only found in culture supernatants, independent of rhomboid background (Fig. S4C and S9A), traveling at a slightly higher molecular mass reflecting the banding pattern of an inactive GlpG-cleaved VesB product. When expression was induced with IPTG, VesBΔ20 was still fully secreted but also auto-activated, similar to WT VesB expressed in a *rssP*::kan mutant strain or with a VesB construct resistant to RssP cleavage (VesB-YY) (Fig. S4D). We next tested if the VesBΔ20 construct exclusively released to the culture supernatant has the potential to be activated by WT VesB. Sterile supernatants from cells expressing VesBΔ20 were incubated with small amounts of sterile supernatants from cells expressing WT VesB, and the activity of VesBΔ20 was probed over time (Fig. S9B). The results show that a small amount of active WT VesB can activate VesBΔ20 in culture, suggesting that VesB cleaved by GlpG has the potential to be activated by RssP-cleaved VesB under native conditions.

To assess OMV localization of VesB variants with and without an RssP-cleavable GlyGly-CTERM, WT VesB, VesBΔ20, VesB-YY, and VesB-HybA were expressed in the Δ*vesABC* mutant strain, and filter sterilized culture supernatants were subjected to ultracentrifugation. The isolated crude OMVs were probed for the presence and activity of VesB. As shown in Fig. 5C, WT VesB had significantly higher activity in the pelleted fraction of culture supernatants compared to the mutant VesB constructs that are not cleaved by RssP despite similar levels of total supernatant activity. These results are reflected in the corresponding Western blot, where relatively similar levels of VesB are detected in the total supernatant. At the same time, WT VesB is the only construct with a prominent band in the pelleted fraction despite equal levels of OmpU detected in the total supernatant and pelleted supernatant (Fig. 5C). Comparable levels of VesB were also detected in the cleared supernatant fractions as determined by protease activity and Western blot, except for VesB-YY, but not OmpU as determined by Western blot (Fig. S8C). As previously mentioned, we hypothesize that this is due to the decrease in stability of this construct.

## DISCUSSION

**Fig 6:**
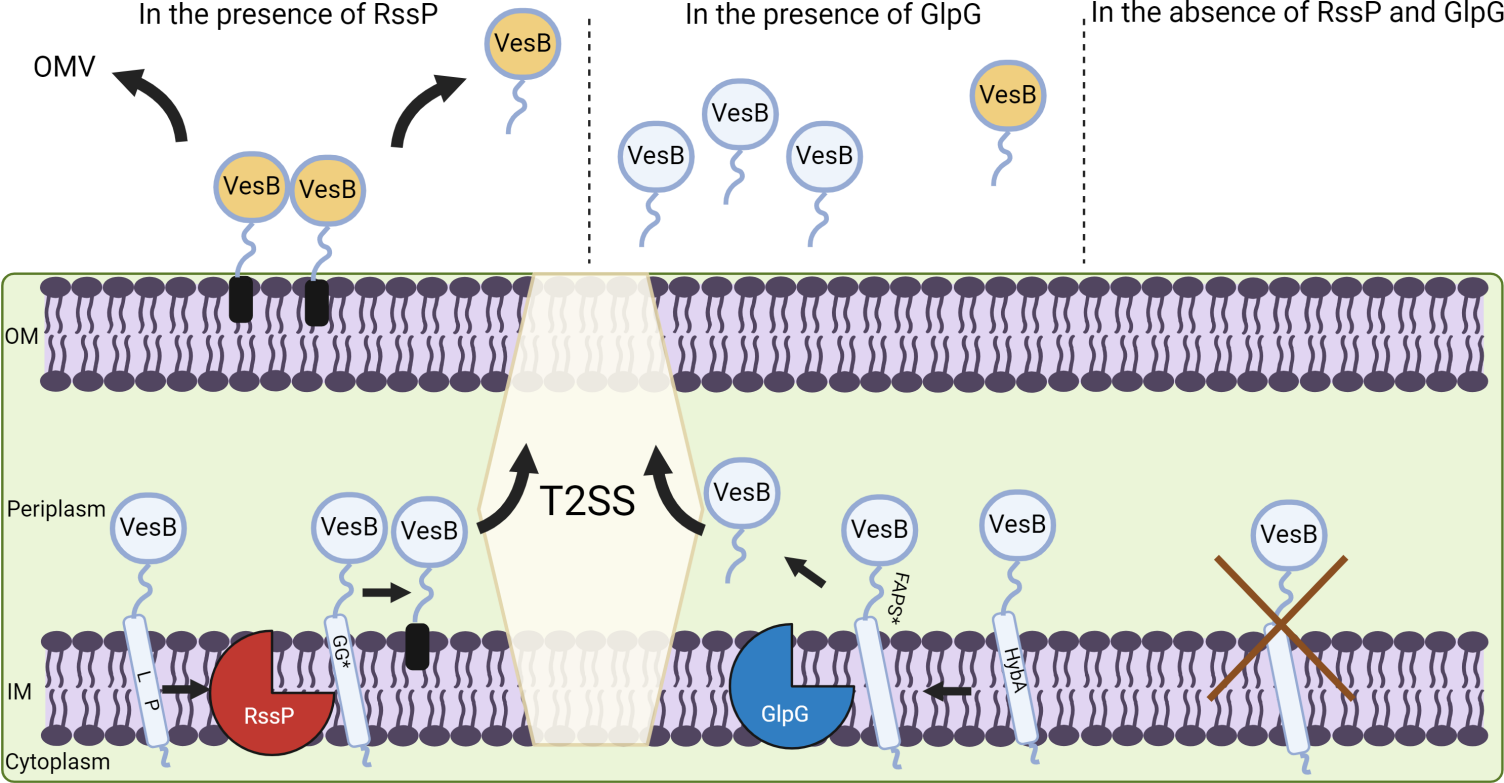
Biogenesis of VesB in *V. cholerae*. With the aid of its signal peptide, VesB is first transported from the cytoplasm to the periplasm via the Sec pathway (not shown). The GlyGly-CTERM anchors VesB to the inner membrane (IM) where rhombosortase (RssP) cleaves after G383 resulting in a posttranslational modification of the newly generated C-terminus denoted by the black cylinder addition. Modified VesB is trafficked to the extracellular space by the T2SS followed by surface retention and autoactivation denoted by the color change. A fraction of VesB is then released to the extracellular space either as a soluble protein or in association with outer membrane vesicles. Cleavage by RssP requires both a conserved leucine residue and a helix-breaking residue, proline in the TMD. Alternatively, in the absence of RssP, GlpG (or a VesB chimera that contains the TMD from HybA) cleaves VesB at an alternate site resulting in an inactive VesB variant that is freely released to the extracellular space following translocation by the T2SS. Autoactivation of GlpG-cleaved VesB can occur with increased VesB expression or accumulation (denoted by the color change). In the absence of either rhomboid protease, VesB remains cell associated, likely trapped in the inner membrane where it is subject to degradation indicated by an X.

A combination of saturation and site-specific mutagenesis reveals that RssP has unique substrate specificity compared to the universally conserved GlpG, which displays a specificity similar to EcGlpG and AraA. RssP prefers small, hydrophobic residues for efficient cleavage at the VesB P1 and P2 sites, but the typical preferences for bulky hydrophobic amino acids favored by GlpG-like rhomboids at P4 are not conserved, distinguishing RssP cleavage specificity from that of universally conserved GlpG, including *V. cholerae* GlpG. In addition to the cleavage site, RssP and GlpG cleave VesB at different locations relative to the membrane milieu, where RssP cleaves VesB within the membrane and GlpG cleaves VesB in the adjacent disordered loop outside the membrane (Fig. 6). Indeed, a helix-breaking residue (P395) is critical for RssP cleavage of VesB, but not for processing by GlpG. This is consistent with an earlier study on rhomboids demonstrating that when a cleavage site is artificially moved outside the lipid bilayer, helix-breaking residues are no longer required for cleavage (25). The predicted interactions of VesB with RssP and GlpG imply that the TMD of VesB is recognized differently between the two proteases, which may explain the difference in cleavage location. Apparently, VesB is targeted specifically to RssP, even in the presence of GlpG. A highly conserved leucine residue in the TMD, L390, appears to be required for this recognition by RssP. Other leucine residues may be less critical; however, it is interesting to speculate that the leucine-rich TMD of VesB may directly interact with similarly leucine-rich TMDs of RssP (Fig. S1). To contrast the recognition of GlyGly-CTERM TMDs by RssP, a chimeric VesB protein containing the TMD of the GlpG substrate HybA from *S. sonnei* was generated. Swapping of the TMD of VesB with that of HybA resulted in a VesB construct that was specifically processed by GlpG over RssP, demonstrating our ability to convert a protein primarily recognized by RssP to one primarily processed by GlpG (Fig. 6). Even in a mutant strain lacking GlpG, RssP still failed to significantly process VesB-HybA, showing the importance of the specific sequence of GlyGly-CTERM in proteolysis by RssP. We speculate that additional rhomboid protease substrates are explicitly recognized by their cognate protease, even in the presence of other rhomboids. This may be critical for bacteria with more than one rhomboid protease, such as *V. cholerae,* or for eukaryotic organisms that have multiple rhomboid proteases. Overall, we demonstrate, using a native substrate, important features of the catalytic mechanism of rhomboid proteases that have largely only been investigated using artificial substrates, especially for GlpG-like rhomboid proteases.

While GlpG is dispensable, RssP is required for the surface localization of VesB. RssP-mediated cleavage of VesB also allows for its efficient auto-activation and release through OMVs (Fig. 6). We are beginning to uncover the physiological role of RssP, which appears to have a direct connection with a group of identified and putative T2SS substrates that may contribute to pathogenesis (18, 26–28). Further study into the GlyGly-CTERM/RssP system will be beneficial to understanding the pathogenesis of bacteria, such as *V. cholerae*, and has the potential to be developed as a cell surface targeting technology. Phylogenic analysis has shown that RssP-like rhomboid proteases co-distribute with GlyGly-CTERM proteins and cluster separately from GlpG-like rhomboids (12). Additional C-terminal sorting motifs were identified in a recent study (29). This study indicated a role for glutamic-type intramembrane endopeptidase homologs, which are also involved in protein sorting in eukaryotes but poorly understood in bacteria (29). Studying both GlyGly-CTERM and other novel C-terminal sorting tags, and their unique sorting enzymes, will provide unique insights into the role these motifs play in bacteria and may demonstrate conserved mechanisms with eukaryotic and archaeal organisms. We show in this study that RssP-like rhomboid proteases have distinct characteristics at the molecular level, and we speculate that these proteases represent a unique subgroup of specialized bacterial rhomboid proteases. We previously have shown that VesB is modified with a glycerophosphoethanolamine moiety that may represent phosphatidylethanolamine through a yet-to-be determined mechanism, which results in its surface localization (10). We speculate that this modification requires prior cleavage of GlyGly-CTERM by RssP, but not GlpG. While further studies are required to verify the modification event, a posttranslational modification at an exposed C-terminal glycine following RssP-mediated proteolysis is reminiscent of the phosphatidylethanolamine modification of the C-terminal glycine of Atg8 (30).

While we show that GlpG cleaves VesB, albeit with lower efficiency than RssP, the physiological role of GlpG proteolysis remains an open question. It is possible that there are growth conditions or environmental factors that favor a soluble rather than surface-bound VesB, and under those conditions, the level of expression of GlpG may be increased or RssP expression diminished. GlpG was initially discovered in *E. coli*, and later in *Salmonella enterica* serovar Typhimurium, where it is expressed from an operon along with GlpE and GlpR that is regulated by glycerol-3-phosphate (31), and investigation into GlpG’s regulation may shed light on its role in VesB cleavage. In addition, two studies using RNA-seq have demonstrated that *rssP* is not regulated by quorum sensing or environmental cues experienced during infection, but instead is constitutively expressed (32, 33). Conversely, in the same studies, *glpG* expression is increased at higher cell density and appears to be regulated by quorum sensing. It is still possible that VesB is an accidental substrate of GlpG. If this is the case, it should not lessen the importance of our study as it has informed us about the specificity of GlpG that can assist in the identification of GlpG’s natural substrates and its physiological role in future studies. The data presented here could fit into a more general role for GlpG encompassing maintenance of misfolded, aggregated, or orphan proteins as demonstrated for SsGlpG (9). Perhaps VesB becomes a substrate of GlpG when it begins to accumulate in the inner membrane and subsequent release of VesB proves to be advantageous to the bacterium under certain growth conditions. Investigating the effect of GlpG on cell growth under various stress-inducing conditions might further illuminate its biological role. The study of rhomboid proteases has been hindered by a lack of known substrates, particularly in bacteria. Several approaches have been used to probe for substrates, including genetic screens and bioinformatic approaches based on substrate characteristics, with limited success (8, 9). We believe that by leveraging findings from this study on VesB, the shared substrate of RssP and GlpG, we are now in a position to identify additional substrates for these proteases and to begin elucidating their overall biological roles in bacteria.

## MATERIALS AND METHODS

### Bacteria strains and plasmids

The *V. cholerae* strain El Tor O1 strain, N16961, and the previously constructed isogenic mutants NΔ*vesABC*, NΔ*glpG*, and NΔ*rssP*::kan were used in this study (10, 18). NΔ*vesABC*Δ*glpG* and NΔ*vesABCrssP*::kan were generated as described using previously published plasmids (10, 18). All plasmids and primers used in this study are listed in Table S1. All polymerase chain reactions (PCR), cloning, and restriction enzyme digestions were done with either SuperFi Platinum polymerase or PfuTurbo, T4 DNA ligase, and restriction enzymes from New England Biolabs and primers that were synthesized at IDT Technologies. pCR-Script (Stratagene) and pMMB67EH constructs were transformed into *E. coli* MC1061 and pCVD442 constructs into SY327λpir. Triparental conjugation was performed with a helper strain carrying pRK2013 to transfer plasmids into N16961 and its isogenic mutants. Overhanging primers coding for the TMD of HybA were used to make the VesB-HybA chimera. Site-specific mutants were generated using overlapping primers and PfuTurbo followed by DpnI digestion and transformation into MC1061 per manufacturer’s instruction.

Site-saturation libraries were prepared using primers containing degenerative codons (NNK) in a two-step PCR setup as previously described (34). Briefly, an initial 500 bp mega primer was synthesized, after which the entire plasmid containing VesB (pMMB67EH) was synthesized using SuperFi Platinum polymerase and transformed into MC1061 after 6 h of DpnI digestion and T4 ligation. As controls, reactions without ligase were performed. Colonies obtained without T4 ligation represented the background WT plasmid at less than 10% of the number of colonies formed with ligation. As proof of concept, 100 colonies at each mutated site were pooled, plasmid DNA was extracted, and Sanger sequencing was performed to detect heterogenetic peaks at the desired positions. In addition, for position 384, 100 colonies were sequenced at random to identify the amino acid coverage. Of the 20 amino acids, 12 unique residues were recovered with less than 20% returning as WT VesB.

### Growth conditions

Strains were grown on Luria-Bertani (LB) agar/broth (Fisher) with 100 µg/mL of carbenicillin (Sigma) when plasmids were present unless otherwise indicated. IPTG was added for the induction of expression where indicated. For analysis of RssP cleavage of VesB, IPTG was omitted, while IPTG was added to observe plasmid-expressed VesB OMV localization or GlpG cleavage of VesB.

### Sodium dodecyl sulfate-polyacrylamide gel electrophoresis and immunoblotting

Samples were prepared and analyzed by sodium dodecyl sulfate-polyacrylamide gel electrophoresis and immunoblotting as described previously (35). Polyclonal antiserum against VesB was incubated with culture supernatant from the Δ*vesABC* mutant for 1 h to pre-absorb cross-reactive antibodies before incubating with the nitrocellulose membrane for 2 h (1:5,000) (36). OmpU antibodies (a kind gift from Karl Klose) were used at 1:20,000 and incubated for 2 h (10). Horseradish peroxidase-conjugated goat anti-rabbit immunoglobulin G (Bio-Rad) used at 1:20,000 was incubated with the membrane for 1 h. Membranes were developed using ECL 2 Western blotting reagent (Thermo Fisher) and visualized using a Typhoon V variable mode imager system and ImageJ imager software.

### Protease assay

*V. cholerae* supernatants and intact cells were measured for protease activity using N-tert-butoxycarbonyl-Gln-Ala-Arg-7-amido-4-methylcoumarin as described previously (36). Change in fluorescence per minute was calculated and converted to moles of methylcoumarin (AMC) generated per minute via a standard curve with known concentrations of AMC. The rate of AMC generation was normalized by OD_600_ of the cultures. For activation of VesBΔ20 culture supernatants, sterile-filtered supernatants from cells expressing VesBΔ20 were combined with 2.5% sterile supernatants from cells expressing WT VesB. Samples were collected at indicated time points and assayed for protease activity. Sterile supernatants from cells expressing VesB-S221A were used as a control, where experiments were performed in biological duplicate and technical triplicate.

### Crude outer membrane vesicle preparation

Cells were grown overnight in LB supplemented with 10 µM IPTG when plasmid was present before culture supernatants were isolated by centrifugation at 10,000 × *g* for 10 minutes. Subsequent sterile filtration and centrifugation at 200,000 × *g* for 3 h pelleted the insoluble fraction. Separately, the cleared supernatant and the pellets containing crude outer membrane vesicles resuspended in LB were assayed for protease activity using the fluorogenic peptide N-tert-butoxycarbonyl-Gln-Ala-Arg-7-amido-4-methylcoumarin and Western blotting using anti-VesB antibodies as described above. For blots from strains natively expressing VesB, 10× concentrated samples were loaded to visualize VesB in OMV fractions.

### Cell surface detection of VesB

Cells were washed, blocked with 2% BSA, and incubated with 1:1,000 of VesB antiserum that was pre-incubated with Δ*vesABC* cells to remove cross-reactive antibodies. Following incubation with 1:1,000 of Alexa Fluor 488 F(ab′)2 goat anti-rabbit immunoglobulin G (Invitrogen) and washing, fluorescence was measured (Ex 488 nm/Em 525 nm). The results were normalized to the fluorescence intensity of the same strain carrying an empty vector as described (10).

### Structural analysis and sequence alignment

Structural models of the interaction of VesB and RssP or GlpG were generated using AlphaFold2 pipeline as implemented in ColabFold (37). Models were generated using AlphaFold2 weights monomer-ptm, multimer-v1, multimer-v2, and multimer-v3 (20). Structures were visualized with Chimera (38). For the GlpG cleavage site sequence logos, a cutoff for the GlyGly-CTERM was determined using the predicted AlphaFold2 structures of VCA0803, VC1200, and VC1649, where the cutoff was made starting in the predicted disordered region, immediately after the folded protease domain, IG-fold, and carbohydrate-binding domain of VesA, B, and C, respectively. For reference, the solved structures of VesB (4LKY) and VesC (6BQM) were used to further determine the IG and carbohydrate-binding domain cutoffs (36, 39). The protein sequences were queried using the NCBI clustered BLAST search. Representative sequences shown in Table S2 were used to generate the sequence logo using the Berkley WebLogo online tool. For the rhomboid protease alignment, sequences were retrieved from NCBI and aligned using ClustalOmega (40).

### Statistical analysis

All data are presented as mean ± standard deviation. The enzyme assays were performed with ≥3 biological replicas in technical triplicates. Differences between the two groups were determined by paired two-tailed Student’s *t*-test. One-way ANOVA with a Dunnett correction for multiple comparisons was used to compare three or more mutants to specified controls. Tukey correction was used for multiple comparisons of three or more samples where every mean was compared to every other mean. Results yielding a *P* value of <0.05 were considered statistically significant. All calculations were done using GraphPad Prism version 10.0.0 for Windows, GraphPad Software, Boston, MA, USA; https://www.graphpad.com).

## SUPPLEMENTAL MATERIAL

Supplemental material mbio.01270-24-s0001.pdf Supplemental tables and figures.

ASM does not own the copyrights to Supplemental Material that may be linked to, or accessed through, an article. The authors have granted ASM a non-exclusive, world-wide license to publish the Supplemental Material files. Please contact the corresponding author directly for reuse.
