# Supplementary material for: Differential processing of VesB by two rhomboid proteases in Vibrio cholerae
Source: mBio. 2024 Aug 13;15(9):e01270-24. doi: 10.1128/mbio.01270-24 (PMC11389362; doi:10.1128/mbio.01270-24)
Supplement: Supplemental material — Supplemental tables and figures. [file mbio.01270-24-s0001.pdf]

Supplemental Material

Supplementary Tables

Sup. Table 1. List of primers and plasmids used.

Gene or Plasmid	Genotypes	Fwd primer 5' to 3'	Rev primer 5' to 3'	RE Sites
pCRScript	cloning vector, AmpR			
pMMB67	Ptac promoter, AmpR			
pCVD442	ori R6K mobRP4 sacB AmpR			
vesB Upstream		GCGGAGCTCCCAAACCTAGTCATGGC	CCACAATGGAGCCTAGAAACCTGATGCACAACCTC	SacI
VesB Downstream		GTGCATCAGGTTTCTAGGCTCCATTGTGGATGAGACGG	CGCCCCGGGGATTTCTCTGGCATG	XmaI
pMMB01	pMMB Fwd Primer	TGGCTGTGCAGGTCGTAAATCAC		
pMMB02	pMMB Rev Primer	TACTCAGGAGAGCGTTCACCGACAAACAAC		
VesBTMDHybA	VesB-HybA chimera	GCAGTGCTGGCTGGTTTGACAGTGCTAGTTAGACGGAAAACCTCTTGA	TAATGGCAGCATCATACCTTTGTACAGTGTGGAACCGCCAGAAGAGGC	
GlpG-S204A		GCGCGAATTTTGGAGGCCTAGCCGGTGTTGTC	GACAACACCGGCTAGGCCTCCAAAATTCGCGC	
VesB-L390V		CGGCTGGTTTGGTGTGCTGCTTTTGG	CCAAAAGCAGCACACCAAACCAGCCG	
VesB-L391V		CGGCTGGTTTGGTTTGGTGCTTTTGGCTCC	GGAGCCAAAAGCACCAAACCAAACCAGCCG	
VesB-L392V		GGTTTGGTTTGCTGGTTTTGGCTCCATTGTGG	CCACAATGGAGCCAAAACCAGCAAACCAAACC	
VesB-L393V		GGTTTGGTTTGCTGCTTGTGGCTCCATTGTGG	CCACAATGGAGCCACAAGCAGCAAACCAAACC	
VesB-G389V		CCATCGGCTGGTTTGTTTTGCTGCTTTTGGCTC	GAGCCAAAAGCAGCAAAACAAACCAGCCGATGG	
VesB-P395V		GGTTTGCTGCTTTTGGCTGTATTGTGGATGAGACGG	CCGTCTCATCCACAATACAGCCAAAAGCAGCAAACC	
VesB-L396V		CCGTCTCATCCACACTGGAGCCAAAAGCAGC	GCTGCTTTTGGCTCCAGTGTGGATGAGACGG	
VesB-Multi	L391V+L392V+L393V+L396V	GGCTGGTTTGGTTTGGTGGTTGTGGCTCCAGTGTGGATGAGACGGA	TCCGTCTCATCCACACTGGAGCCACAACCACCAAACCAAACCAGCC	
VesBΔ373-379	delS373-A379	CGTCTCGTATTCAACTGGATACTTCTTCTGGCGGT	ACCGCCAGAAGAAGTATCCAGTTGAATACGAGACG	
VesB-AA	G382A+G383A	GCCTCTTCTGCCGCTTCCATCGGCTG	GCCGATGGAAGCGGCAGAAGAGGCA	
VesB-YY	G382Y+G383Y	CCTTTTGCCCCAAGTGCCTCTTCTTATTATTCCATCGGCTGGTTTGGTTTGCT	AGCAAACCAAACCAGCCGATGGAATAATAAGAAGAGGCACTTGGGGCAAAAGG	
VesB-S378X	site saturation	GAAAGATTCATGTCAGGGAGATAGTGGTGGCCCAATTGTAG	CCAGAAGAGGCMNNTGGGGCAAAAGGCGAAGTATC	
VesB-A379X	site saturation	GAAAGATTCATGTCAGGGAGATAGTGGTGGCCCAATTGTAG	GGAACCGCCAGAAGAMNNACTTGGGGCAAAAGGC	
VesB-S380X	site saturation	GAAAGATTCATGTCAGGGAGATAGTGGTGGCCCAATTGTAG	GAACCGCCAGAMNNGGCACTTGGGGCA	
VesB-S381X	site saturation	GAAAGATTCATGTCAGGGAGATAGTGGTGGCCCAATTGTAG	GATGGAACCGCCMNNAGAGGCACTTGGGGCAAAAGGCGAAG	
VesB-G382X	site saturation	GAAAGATTCATGTCAGGGAGATAGTGGTGGCCCAATTGTAG	GCCGATGGAACCMNNAGAAGAGGCACTTGGGGCAAAAGG	
VesB-G383X	site saturation	GAAAGATTCATGTCAGGGAGATAGTGGTGGCCCAATTGTAG	GCCGATGGAMNNGCCAGAAGAGGCACTTGGGG	
VesB-S384X	site saturation	GAAAGATTCATGTCAGGGAGATAGTGGTGGCCCAATTGTAG	AAACCAGCCGATMNNACCGCCAGAAGAGGCACTTG	
VesB-I385X	site saturation	GAAAGATTCATGTCAGGGAGATAGTGGTGGCCCAATTGTAG	CAAACCAAACCAGCCMNNGGAACCGCCAGAAGAG	
VesB-G386X	site saturation	GAAAGATTCATGTCAGGGAGATAGTGGTGGCCCAATTGTAG	GCAAACCAAACCAMNNGATGGAACCGCC	
VesB-W387X	site saturation	GAAAGATTCATGTCAGGGAGATAGTGGTGGCCCAATTGTAG	AGCAGCAAACCAAAMNNGCCGATGGAACCGCC	
VesB-F375A		TCGTATTCAACTGGATACTTCGCCTGCTGCCCCAAGTGCC	GGCACTTGGGGCAGCAGGCGAAGTATCCAGTTGAATACGA	
VesB-F375V		CTGGATACTTCGCCTATTGCCCCAAGTGCCT	AGGCACTTGGGGCAATAGGCGAAGTATCCAG	
VesB-F375I		CTGGATACTTCGCCTGTTGCCCCAAGTGCCT	AGGCACTTGGGGCAACAGGCGAAGTATCCAG	
VesB-A376F		CGTATTCAACTGGATACTTCGCCTTTTTTCCCAAGTGCCTCTT	AAGAGGCACTTGGGAAAAAAGGCGAAGTATCCAGTTGAATACG	
VesB-P377F		GGATACTTCGCCTTTTGCCTTCAGTGCCTCTTCTGGCGGTT	AACCGCCAGAAGAGGCACTGAAGGCAAAAGGCGAAGTATCC	
VesB-S378F		TGGATACTTCGCCTTTTGCCCCATTTGCCTCTTCTGGC	GCCAGAAGAGGCAAATGGGGCAAAAGGCGAAGTATCCA	
VesB-A379F		GCCTTTTGCCCCAAGTTTCTCTTCTGGCGGTTCC	GGAACCGCCAGAAGAGAAACTTGGGGCAAAAGGC	
VesB-A379E		CCTTTTGCCCCAAGTGAGTCTTCTGGCGGTTCCA	TGGAACCGCCAGAAGACTCACTTGGGGCAAAAGG	
VesB-A379D		CTTTTGCCCCAAGTGACTCTTCTGGCGGTTC	GAACCGCCAGAAGAGTCACTTGGGGCAAAAG	
VesB-S380F		GCCCCAAGTGCCTTTTCTGGCGGTTCC	GGAACCGCCAGAAAAGGCACTTGGGGC	
VesB-S381H		CCTTTTGCCCCAAGTGCCTCTCATGGCGGTTCCA	TGGAACCGCCATGAGAGGCACTTGGGGCAAAAGG	
VesB-S381I		CCTTTTGCCCCAAGTGCCTCTATTGGCGGTTCCA	TGGAACCGCCAATAGAGGCACTTGGGGCAAAAGG	
VesB-S381M		CGCCTTTTGCCCCAAGTGCCTCTATGGGCGGTTCCATC	GATGGAACCGCCCATAGAGGCACTTGGGGCAAAAGGCG	
VesB-S381V		CCTTTTGCCCCAAGTGCCTCTGTTGGCGGTTCCA	TGGAACCGCCAACAGAGGCACTTGGGGCAAAAGG	

VesB-S381W		TTGCCCCAAGTGCCTCTTGGGGCGGTTCCATC	GATGGAACCGCCCCAAGAGGCACTTGGGGCAA
VesB-S381Y		TGCCCCAAGTGCCTCTTATGGCGGTTCC	GGAACCGCCATAAGAGGCACTTGGGGCA
VesB-G382C		CCCCAAGTGCCTCTTCTTGCGGTTCCATC	GATGGAACCGCAAGAAGAGGCACTTGGGG
VesB-G382E		CAAGTGCCTCTTCTGAGGGTTCCATCGGCTGG	CCAGCCGATGGAACCCTCAGAAGAGGCACTTG
VesB-G382F		CCCCAAGTGCCTCTTCTTTCGGTTCCATCGGC	GCCGATGGAACCGAAAGAAGAGGCACTTGGGG
VesB-G382H		CCCCAAGTGCCTCTTCTCACGGTTCCATCGGC	GCCGATGGAACCGTGAGAAGAGGCACTTGGGG
VesB-G382I		CCCCAAGTGCCTCTTCTATCGGTTCCATCGGC	GCCGATGGAACCGATAGAAGAGGCACTTGGGG
VesB-G382W		CCCAAGTGCCTCTTCTTGGGGTTCCATCGGCT	AGCCGATGGAACCCCAAGAAGAGGCACTTGGG
VesB-G382V		CAAGTGCCTCTTCTGTCGGTTCCATCGGCTG	CAGCCGATGGAACCGACAGAAGAGGCACTTG
VesB-G383C		CCAGCCGATGGAACAGCCAGAAGAGGCAC	GTGCCTCTTCTGGCTGTTCCATCGGCTGG
VesB-G383E		CAAACCAGCCGATGGACTCGCCAGAAGAGGCACT	AGTGCCTCTTCTGGCGAGTCCATCGGCTGGTTTG
VesB-G383F		CAAACCAGCCGATGGAAAAGCCAGAAGAGGCACTTG	CAAGTGCCTCTTCTGGCTTTTCCATCGGCTGGTTTG
VesB-G383Q		CCAAACCAGCCGATGGACTGGCCAGAAGAGGCACTTG	CAAGTGCCTCTTCTGGCCAGTCCATCGGCTGGTTTGG
VesB-G383W		CAAACCAGCCGATGGACCAGCCAGAAGAGGCACTT	AAGTGCCTCTTCTGGCTGGTCCATCGGCTGGTTTG
VesB-S384C		CAAACCAGCCGATGCAACCGCCAGAAGAG	CTCTTCTGGCGGTTGCATCGGCTGGTTTG
VesB-S384D		CCAAACCAGCCGATGTCACCGCCAGAAGAGGC	GCCTCTTCTGGCGGTGACATCGGCTGGTTTGG
VesB-S384E		CAAACCAAACCAGCCGATCTCACCGCCAGAAGAGGCACT	AGTGCCTCTTCTGGCGGTGAGATCGGCTGGTTTGGTTTG
VesB-S384K		CAAACCAAACCAGCCGATCTTACCGCCAGAAGAGGCACT	AGTGCCTCTTCTGGCGGTAAGATCGGCTGGTTTGGTTTG
VesB-S384V		CCAAACCAGCCGATGACACCGCCAGAAGAGGC	GCCTCTTCTGGCGGTGTCATCGGCTGGTTTGG
VesBΔ20	deletion of final 20 AA after G383	TGATTACCTATCCGAGATCTGCAGGGGCAG	ACCGCCAGAAGAGGCACTTGGGG
HybA G->V		GTTCCACACTGTACAAAGTTATGATGCTGCCATTAGC	GCTAATGGCAGCATCATAACTTTGTACAGTGTGGAAC
HybA G->R		GGTTCCACACTGTACAAACGTATGATGCTGCCATTAG	CTAATGGCAGCATCATACGTTTGTACAGTGTGGAACC

Sup. Table 2. List of representative sequences used to generate sequence logo for VesA, B and C.

VesA	VesB	VesC
CSB76112.1	EGR0443395.1	WP_199357358.1
MVE67853.1	HAV0075612.1	MVE89208.1
CSB66271.1	CSB94426.1	CSB21702.1
CSA60582.1	MCX9553579.1	EJH71155.1
WP_113628564.1	CSB59105.1	WP_229764901.1
WP_218762997.1	WP_032481011.1	WP_053044174.1
MCX9564208.1	WP_267789668.1	WP_229598643.1
WP_005525620.1	WP_001043974.1	CSA34250.1
WP_140418008.1	WP_219659064.1	WP_032468785.1
	WP_154401439.1	WP_217012061.1
	WP_113690374.1	WP_199355353.1
	WP_085604026.1	WP_002041620.1
	HDQ5351420.1	WP_089071506.1
	WP_142578324.1	WP_033928381.1
	EAZ75563.1	WP_005513644.1
	WP_057642314.1	
	WP_199353927.1	
	WP_276114421.1	
	GHW56943.1	
	CSB12135.1	
	GFK33235.1	
	WP_069648103.1	
	WP_142737935.1	
	RND30057.1	
	WP_032468015.1	

Supplementary Figures

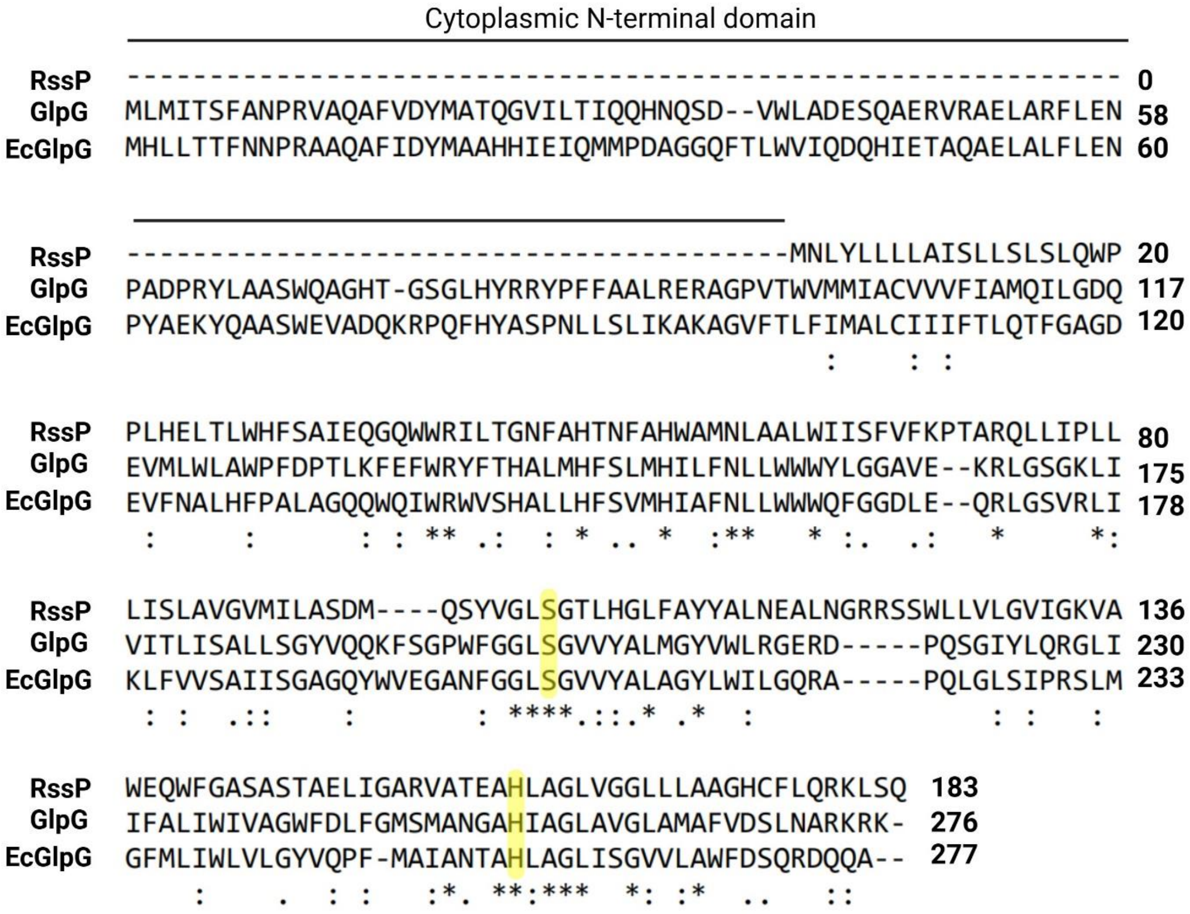

Figure S1. Sequence alignment of the rhomboid proteases from *V. cholerae.* Amino acid sequences of RssP, GlpG, and EcGlpG (VC1981, VC0099, and CAD6000690.1 respectively) were retrieved from NCBI and aligned using ClustalOmega. The residues making up the catalytic dyad are highlighted in yellow. The soluble N-terminal domain is indicated. Conservation is indicated with * representing completely conserved positions and . , : indicate various levels of conservation.

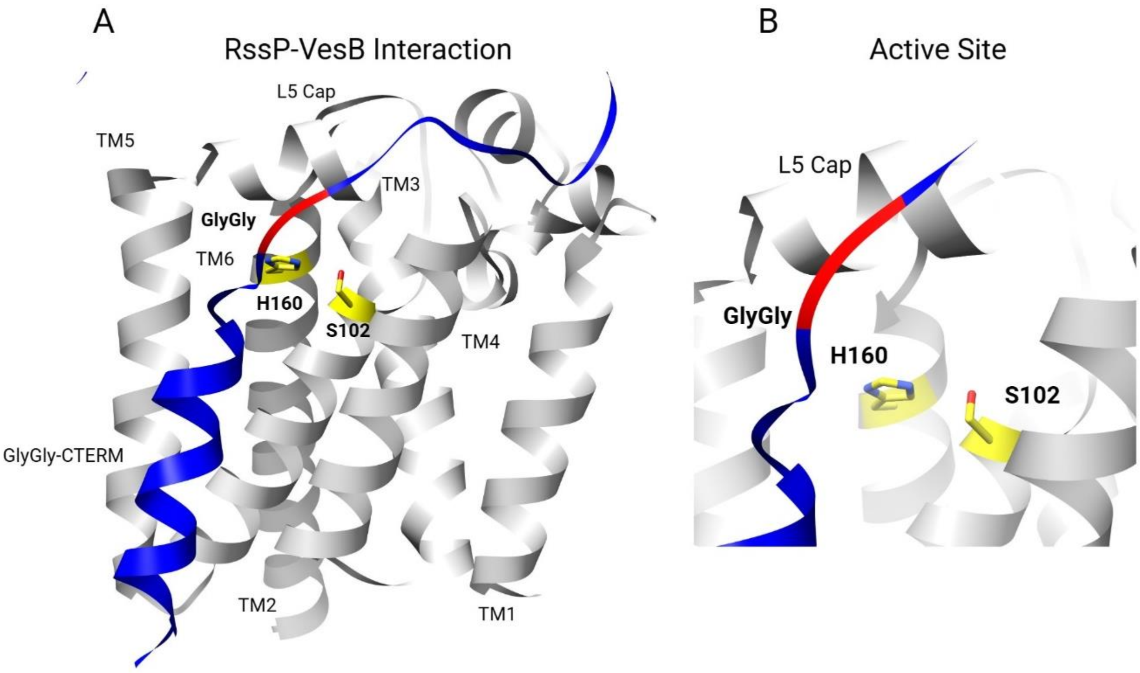

Figure S2. Predicted interaction of RssP and VesB representing a potential docking confirmation. Predicted AlphaFold2 interaction of RssP (grey) with VesB (blue) is shown with TMDs, L5 cap, and catalytic residues (yellow) of RssP and GlyGly of VesB (red) indicated (**A**). Close up of RssP active site is shown (**B**). Predicted alignment error is shown in **Sup. Fig 3B**.

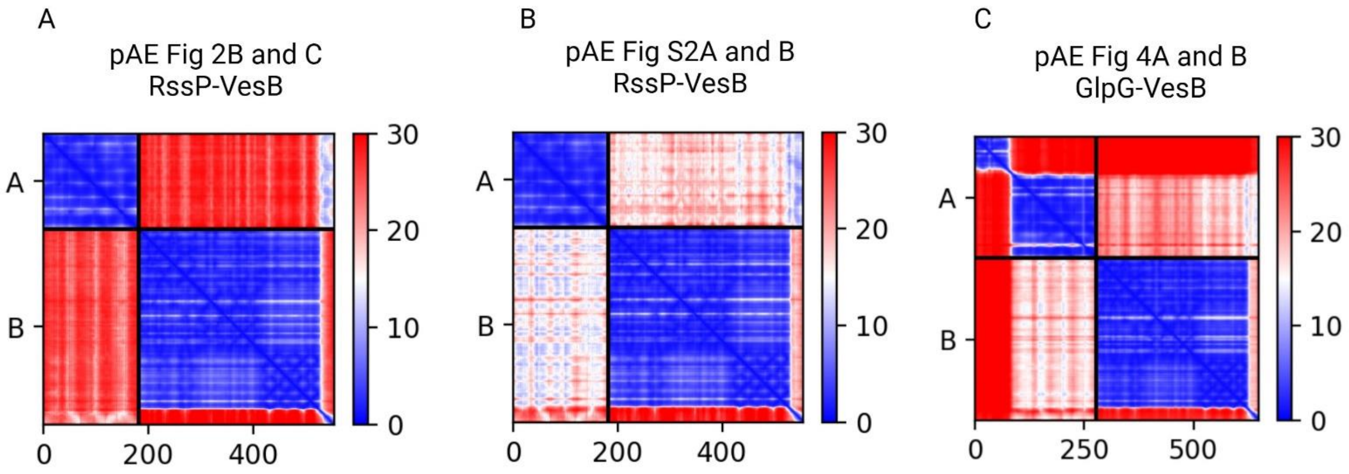

Figure S3. Predicted alignment error of RssP and GlpG interaction with VesB. A, B. AlphaFold2 was used to generate the interaction between RssP and VesB and the predicted alignment error is shown. **C.** AlphaFold2 was used to generate the interaction between GlpG and VesB with predicted alignment error shown. For each plot A designates the respective rhomboid protease and B designates VesB. The predicted alignment error for the given figures is presented with values ranging from 0 Å (blue) to 30 Å (red).

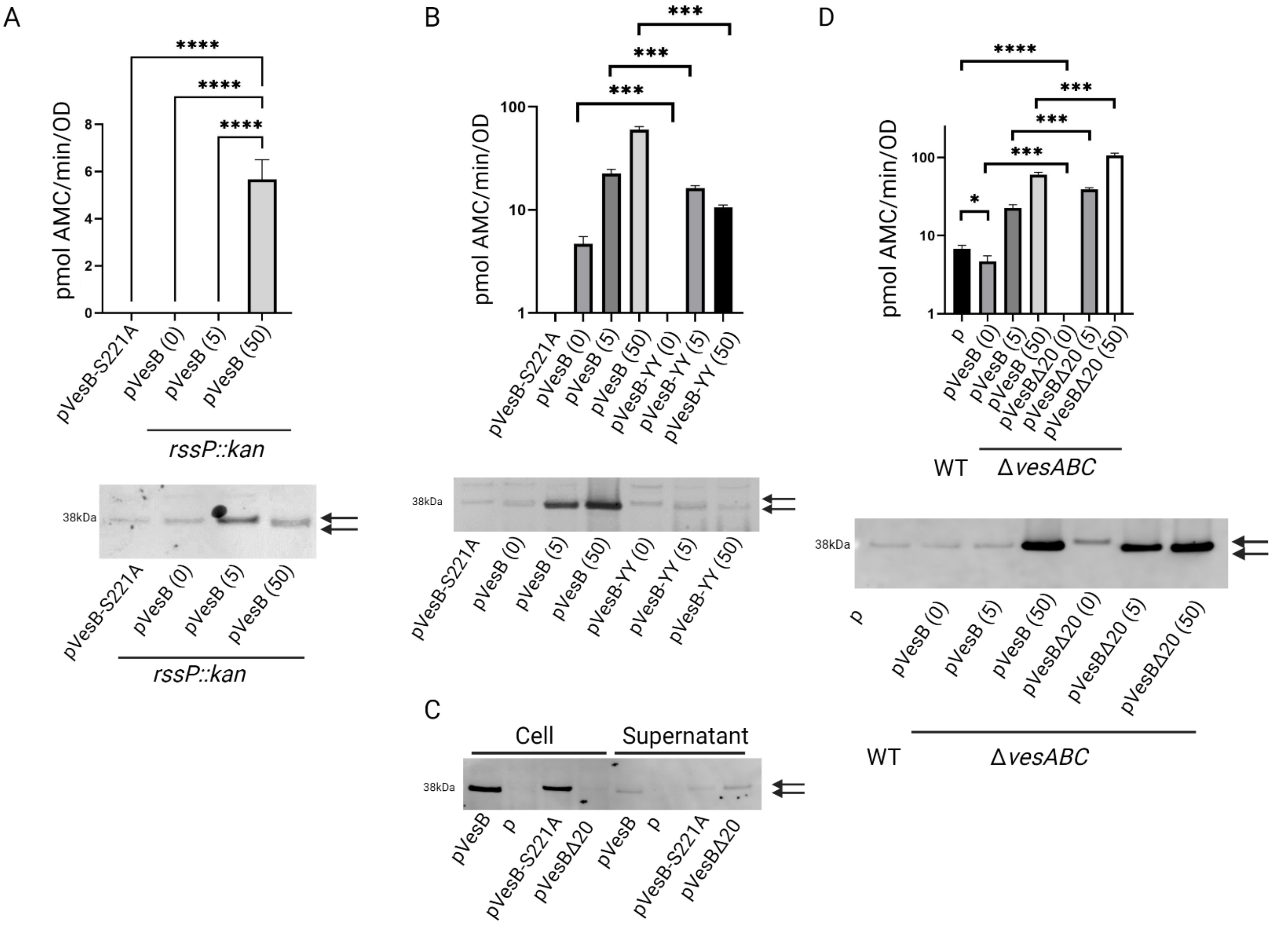

Figure S4. GlpG-cleaved VesB is not as efficiently activated as RssP-cleaved VesB. A. Culture supernatants from the Δ*vesABC rssP::kan* mutant strain expressing WT VesB grown with increasing amounts of IPTG (μM) were assessed for serine protease activity using the fluorogenic peptide Boc-Gln-Ala-Arg-AMC (mean ± SD of n=3)**.** ****P<0.0001 by one-way ANOVA analysis with Dunnette multiple comparisons test. Samples were also run on SDS-PAGE, transferred to nitrocellulose membrane, and blotted against anti-VesB antibodies. Arrows indicate inactive (top) and active (bottom) VesB. **B.** Culture supernatants of the mutant strain Δ*vesABC* containing empty vector (p) or plasmid harboring indicated VesB constructs were processed as in **A.** Activity is shown in log scale and data represent mean ± SD of n=3 experiments. ***P<0.001 by Student's t-test. Fractions were also run on SDS-PAGE, transferred to nitrocellulose membrane, and blotted with anti-VesB antibodies. **C.** VesBΔ20 construct lacking all residues C-terminal of the GlyGly motif was generated by deleting the *vesB* codons for the last 20 amino acids of VesB. Cultures of the Δ*vesABC* mutant strain containing either empty vector, pVesB, pVesB-S221A, or pVesBΔ20 were separated into cell and supernatant fractions and run on SDS-PAGE, transferred to nitrocellulose membrane, and blotted for VesB. Arrows indicate active (bottom) and inactive (top) VesB. **D.** Supernatants from cultures induced with indicated IPTG concentrations of the Δ*vesABC* mutant strain containing either empty vector, pVesB, or pVesBΔ20 were assessed for serine protease activity using the fluorogenic peptide Boc-Gln-Ala-Arg-AMC (mean ± SD of n=3). *<0.05, ***P<0.001, ****P<0.0001 by Student's t-test. Activity is displayed in log scale. Fractions were also run on SDS-PAGE, transferred to nitrocellulose membrane, and blotted with anti-VesB antibodies. Fractions from IPTG-induced cultures were loaded at one-fourth of the concentration of non-induced cultures to distinguish between active and inactive VesB. For comparison, WT *V. cholerae* with an empty vector (p) was also examined as a control. Arrows indicate inactive (top) and active (bottom) VesB. All activity assays were performed with three biological replicates in technical triplicate. Western blots are shown from at least two blots performed on biological samples.

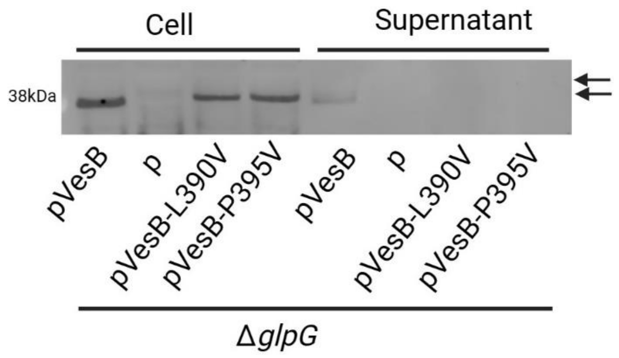

Figure S5. When VesB-L390V and VesB-P395V are expressed in a *glpG* mutant strain they remain associated with the cell. Cultures of the mutant strain Δ*vesABC*Δ*glpG* containing empty vector (p) or plasmids encoding VesB constructs were separated into cell and supernatant fractions and run on SDS-PAGE, transferred to nitrocellulose membrane, and blotted with anti-VesB antibodies.

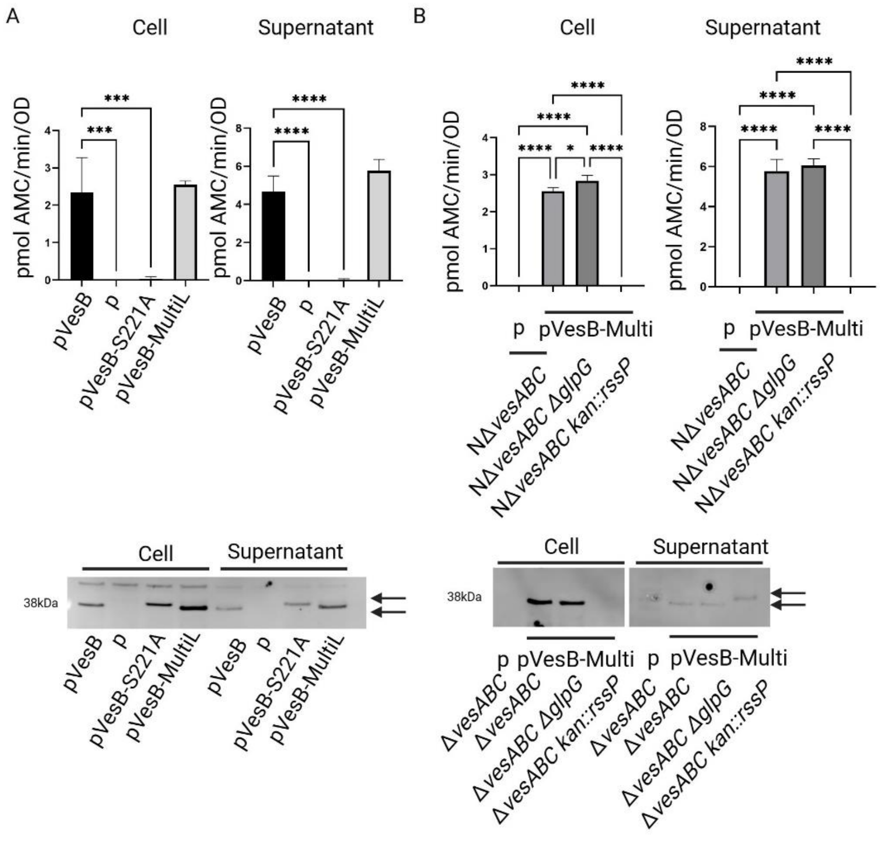

Figure S6. Additional leucine residues in the TMD of VesB are not essential to RssP mediated VesB cleavage. A. Cultures of the Δ*vesABC* mutant strain containing empty vector (p) or plasmid harboring indicated VesB constructs were separated into cell and supernatant fractions and assessed for serine protease activity using the fluorogenic peptide Boc-Gln-Ala-Arg-AMC. Data represent mean ± SD of n=3 experiments with ***P<0.001, ****P<0.0001 by one-way ANOVA analysis with Dunnette multiple corrections test. Fractions were also run on SDS-PAGE, transferred to nitrocellulose membrane, and blotted with anti-VesB antibodies. Arrows indicate active (bottom) and inactive (top) VesB. **B.** VesB-MultiL was ectopically overexpressed (50 µM) in the *ΔvesABC* mutant strain and in the same mutant strain with *glpG* deleted (*ΔglpG*) or *rssP* disrupted (*rssP::kan*). Samples were separated into cell and supernatant fractions and assessed for serine protease activity using the fluorogenic peptide Boc-Gln-Ala-Arg-AMC (mean ± SD of n=3). *P<0.05, P< 0.001, ****P<0.0001 by one-way ANOVA analysis with Tukey multiple corrections test. Fractions were also run on SDS-PAGE, transferred to nitrocellulose membrane, and blotted with anti-VesB antibodies. Representative Western blots are shown from at least two blots performed on biological samples.

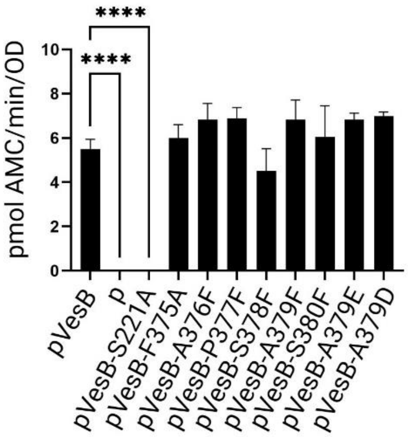

Figure S7. VesB constructs expressed with substitutions of residues important for GlpG-mediated cleavage are still functional in a WT background. Culture supernatants of mutant strain Δ*vesABC* containing an empty vector (p) or plasmid coding for indicated VesB constructs were assessed for serine protease activity using the fluorogenic peptide Boc-Gln-Ala-Arg-AMC. Data represent mean ± SD of n=3 experiments with ****P<0.0001 by one-way ANOVA analysis with Dunnette multiple corrections tests.

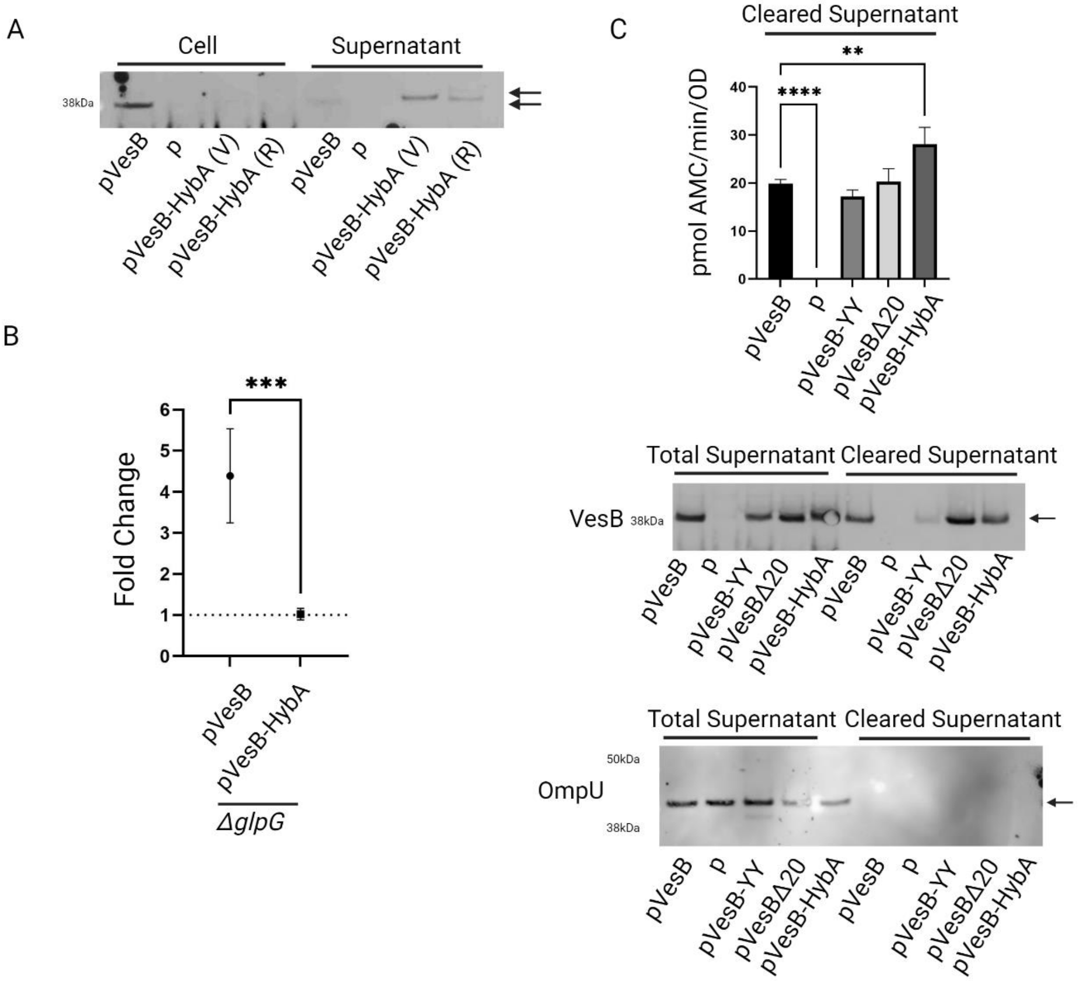

Figure S8. The VesB-HybA chimera is processed similar to other GlpG-cleaved VesB constructs. **A.** Strain Δ*vesABC* containing pVesB or pVesB-HybA with the known HybA P1 residue glycine mutated to arginine (G->R) were separated into cell and supernatant fractions, run on SDS-PAGE, transferred to nitrocellulose membrane, and blotted with anti-VesB antibodies. **B.** WT VesB or the VesB-HybA chimera were ectopically overexpressed (50 µM) in the *ΔvesABCΔglpG* mutant strain. Cell fractions were isolated after growth in M9 media supplemented with casamino acids and glucose and the surface exposed VesB was probed by incubating intact cells with anti-VesB followed by incubation with goat-anti rabbit IgG coupled with ALEXA Fluor 488 and scanning for fluorescence in 96-well format (mean ± SD of n=5). Relative surface amount was determined by comparing fluorescence intensities of the cells of pVesB and pVesB-HybA containing strains to cells of the same strains containing an empty vector. ***P<0.001 by Student t-test. **C.** Supernatants from cultures induced with 10 µM IPTG of mutant strain Δ*vesABC* containing empty vector (p), pVesB, pVesB-YY, pVesBΔ20, or pVesB-HybA were sterile filtered and subjected to high-speed centrifugation to remove crude outer membrane vesicles from the supernatant. Cleared supernatant was analyzed for serine protease activity and Western blotting using antibodies against VesB (top) and OmpU (bottom). **<0.01, ***P< 0.001, ****P<0.0001 by one-way ANOVA analysis with Dunnette multiple corrections test. Representative Western blots are shown from at least two blots performed on biological samples.

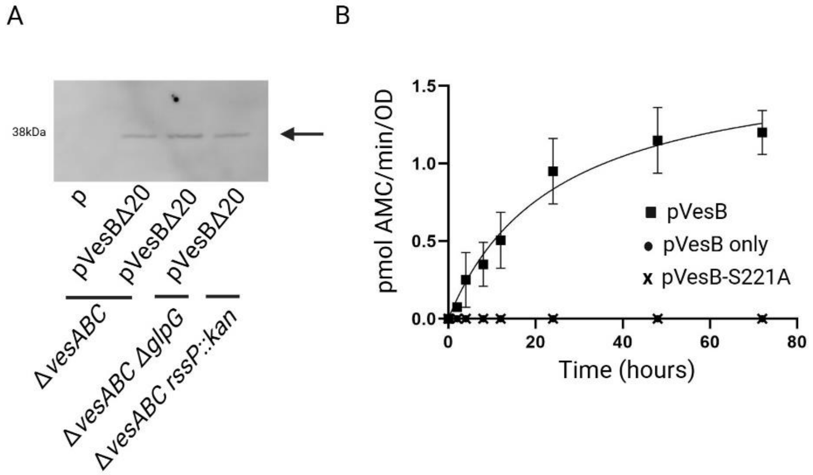

Figure S9. A VesB construct lacking the 20 C-terminal amino acids is fully secreted and can be activated in the presence of WT VesB. A. VesBΔ20 construct lacking all residues C-terminal of the GlyGly motif was generated by deleting the *vesB* codons for the last 20 amino acids of VesB. Cultures of the Δ*vesABC* mutant strains containing empty vector (p) or pVesBΔ20 or mutant strains Δ*vesABC* with *glpG* (*ΔglpG*) deleted or rssP disrupted (*rssP::kan*) containing pVesBΔ20 were separated into cell and supernatant fractions and run on SDS-PAGE, transferred to nitrocellulose membrane, and blotted for VesB. Arrows indicate inactive VesB. **B.** Culture supernatants from strains containing pVesBΔ20 were sterile filtered and incubated with 2.5% sterile filtered supernatant from similarly processed WT VesB containing culture. Fractions were collected at the indicated time points and assessed for serine protease activity using the fluorogenic peptide Boc-Gln-Ala-Arg-AMC (mean ± SD for n=2). Non-linear regression was used to fit the data points (R squared value=0.93). For comparison, sterile supernatants from cells containing VesB-S221A were added to the VesBΔ20 containing supernatant. Another control included the 2.5% WT VesB inoculum alone.
